# Metabolomics-based strategy to assess drug hepatotoxicity and uncover the mechanisms of hepatotoxicity involved

**DOI:** 10.1007/s00204-023-03474-8

**Published:** 2023-04-06

**Authors:** Teresa Martínez-Sena, Erika Moro, Marta Moreno-Torres, Guillermo Quintás, Jan Hengstler, José V. Castell

**Affiliations:** 1 Instituto de Investigación Sanitaria del Hospital La Fe (IIS La Fe), Unidad Mixta de Hepatologia Experimental, Valencia, Spain; 2 Departamento de Química Analítica, Facultad de Químicas, Universidad de Valencia, Valencia, Spain; 3 Departamento de Bioquímica y Biología Molecular, Facultad de Medicina, Universidad de Valencia, Valencia, Spain; 4 Instituto de Salud Carlos III, CIBEREHD, Madrid, Spain; 5 Health and Biomedicine, Leitat Technological Center, Valencia, Spain; 6 Analytical Unit, Health Research Institute La Fe, Valencia, Spain; 7 Leibniz Research Centre for Working Environment and Human Factors at the Technical University of Dortmund (IfADo), Dortmund, Germany

**Keywords:** Metabolomics, Drug hepatotoxicity, Mechanisms of hepatotoxicity, In vitro, HepG2 cells

## Abstract

Toxicity studies, among them hepatotoxicity, are key throughout preclinical stages of drug development to minimise undesired toxic effects that might eventually appear in the course of the clinical use of the new drug. Understanding the mechanism of injury of hepatotoxins is essential to efficiently anticipate their potential risk of toxicity in humans. The use of in vitro models and particularly cultured hepatocytes represents an easy and robust alternative to animal drug hepatotoxicity testing for predicting human risk. Here, we envisage an innovative strategy to identify potential hepatotoxic drugs, quantify the magnitude of the alterations caused, and uncover the mechanisms of toxicity. This strategy is based on the comparative analysis of metabolome changes induced by hepatotoxic and non-hepatotoxic compounds on HepG2 cells, assessed by untargeted mass spectrometry. As a training set, we used 25 hepatotoxic and 4 non-hepatotoxic compounds and incubated HepG2 cells for 24 h at a low and a high concentration (IC10 and IC50) to identify mechanism-related and cytotoxicity related metabolomic biomarkers and to elaborate prediction models accounting for global hepatotoxicity and mechanisms-related toxicity. Thereafter, a second set of 69 chemicals with known predominant mechanisms of toxicity and 18 non-hepatotoxic compounds were analysed at 1, 10, 100 and 1000 µM concentrations from which and based on the magnitude of the alterations caused as compared with non-toxic compounds, we defined a “toxicity index” for each compound. In addition, we extracted from the metabolome data the characteristic signatures for each mechanism of hepatotoxicity. The integration of all this information allowed us to identify specific metabolic patterns and, based on the occurrence of that specific metabolome changes, the models predicted the likeliness of a compound to behave as hepatotoxic and to act through a given toxicity mechanism (i.e., oxidative stress, mitochondrial disruption, apoptosis and steatosis) for each compound and concentration.

**Supplementary Information:**

The online version contains supplementary material available at 10.1007/s00204-023-03474-8.

## Introduction

Liver is the principal organ where drugs tend to reach the highest concentration, or even to accumulate after oral dosage and where they undergo active biotransformation and bioactivation reactions potentially causing liver injury. This explains the susceptibility of this organ to drugs. Hepatotoxicity still is one of the major reasons for drug withdrawal in the preclinical stage (Lee 2003). Besides, drug-induced liver injury (DILI) is also a challenge in pharmacovigilance, as toxic events in polymedicated patients may occur as a result of unforeseen drug–drug interactions (Doan et al. 2013; Khezrian et al. 2020). DILI can be originated from a variety of metabolic initiating and key events which are related to the nature and concentration of the drug, as well as to genetic factors and the exposome of each individual. Although precise and individualised mechanisms of toxicity have been properly identified (Tolosa et al. 2012b), hepatotoxicity is a complex phenomenon where frequently more than one toxicity mechanism acts on hepatocytes.

In the course of the early stages of drug development, in vitro models are used as a fast and cost-affordable strategy for lesser hepatotoxic drug candidate selection. Primary human hepatocytes (PHHs) remain, for this purpose, as the gold standard in in vitro hepatotoxicity studies. However, their variability, cost and in-time accessibility have made other cell systems valuable alternatives. Among them, the human hepatoma HepG2 cells are widely used in hepatotoxicity studies as an easy to handle and robust cell line, circumventing the limitations of availability, reproducibility and cost associated to the use of primary hepatocytes (Donato et al. 2009; Tolosa et al. 2012b; Kamalian et al. 2015). Although these cells show limited drug biotransformation activities and are not best suited to evidence bioactivation-related toxicity phenomena (Castell et al. 2006), unless they are upgraded with adenoviral vectors to overexpress biotransformation activities (Gómez-Lechón et al. 2010; Tolosa et al. 2012a), they display many differentiated hepatic functions and, as such, are targets for direct-acting hepatotoxic compounds interfering with hepatocyte metabolism. Based on that, they are suitable for in vitro testing (Brandon et al. 2003).

Hepatotoxicity is a complex phenomenon that involves a series of initiating molecular events that tend to have diverse and broad consequences on metabolic pathways of exposed cells. For instance, compounds acting as mitochondrial toxins and known to inhibit enzymes of the electron transport chain (Nolfi-Donegan et al. 2020) will impair fatty acid ß-oxidation (FAO) as well (Grünig et al. 2018). This impairment of mitochondrial FAO causes microvesicular steatosis, which can evolve into an inflammatory status, *steatohepatitis*, and further progression to cirrhosis (Farrell and Larter 2006). On the other hand, uncoupling of the electron transport chain can also give rise to a partial reduction of molecular oxygen and the generation of intermediate reactive oxygen species, causing an imbalance in cells known as *oxidative stress* (Pizzino et al. 2017). This is evidenced by decreased levels of reduced glutathione (Kaplowitz 1981; Irie et al. 2016) and the presence of oxidised lipid metabolites.

Apoptosis, unlike necrosis, is independent of ATP depletion in the early stages. However, a high extent of cell damage and mitochondrial disruption (decrease of mitochondrial membrane potential) result in lower ATP levels shifting from apoptosis to necrosis (Jaeschke et al. 2004). Decrease in ATP levels affect indirectly many other hepatocyte functions (urea synthesis, lipid synthesis, plasma protein synthesis, bile acid synthesis and transport) (Labbe et al. 2008; Mansouri et al. 2018). In other words, although each mechanism of toxicity can conceptually be identified as a well differentiated processes with recognisable molecular initiating events leading to structural and functional cell injury (key events), drug hepatotoxicity is caused by a commingled of mechanisms, that tend to overlap each other. Thus, the high interconnection among them increase the difficulty of identifying selective and specific biomarkers for isolated hepatotoxicity mechanism (Fariss et al. 2005).

The recent developments in *‘omics’* technologies, with the help of powerful data analysis tools, offer in-depth information regarding biochemical changes occurring in cells/tissues, as a consequence of the toxic insult, and offer many opportunities for global and mechanism-specific toxicity biomarker identification (Yong et al. 2020). Previous reports have evidenced specific metabolic patterns of toxicity, capable of discriminating among the different toxicity outcomes (Vorkas et al. 2015; Ramirez et al. 2018; Quintás et al. 2021). Preliminary evidence from our laboratory, also showed that HepG2 in combination with metabolomic analysis, could be a good approach for hepatotoxicity investigation (García-Cañaveras et al. 2015). Based on these premises, we further explored the use of ultra-performance liquid chromatography–mass spectrometry based untargeted metabolomics for the characterization of the metabolic changes occurring upon exposure of cells to hepatotoxic and non-hepatotoxic compounds, and to estimate the participation of the different mechanisms of toxicity in the global hepatotoxicity of a given compound.

Selection of a *training set* of compounds acting through the different hepatotoxicity mechanisms was made on the basis of solid bibliographic references of scientific literature, as well as our own expertise, having worked for a long time on drug hepatotoxicity research. Thus, we chose 29 chemicals for which there was a clear consensus about their mode of action and preferential mechanism of hepatotoxicity, and were classified accordingly into five major mechanisms groups, i.e., oxidative stress (OS), mitochondrial disruption (MI), apoptosis (APT), steatosis (ST) and cholestasis (CHOL), (Manivel et al. 1987; Sentürk et al. 2008; Gómez-Lechón et al. 2010; Olayinka et al. 2012; Tolosa et al. 2012b; Afolabi and Oyewo 2014; Stocco et al. 2014; Rodrigues et al. 2018), Table 1. The analysis of cellular metabolomic changes was performed at two concentrations: IC10 to appreciate initial changes in the mechanism of toxicity and to another somewhat higher concentration, IC50, to see reinforced signals although probably overlapping those of general cytotoxicity. An appropriate analysis enabled us to identify the metabolic patterns associated with each specific mechanism of drug-induced hepatotoxicity and that linked to general cytotoxicity. Results were further validated using a *test set* comprising 69 chemicals exerting known hepatotoxicity through a predominant mechanism of toxicity, and 18 non-hepatotoxic compounds (Gómez-Lechón et al. 2010; Tolosa et al. 2012b; Chen et al. 2016). The concentrations tested (1, 10, 100 and 1000 µM) were selected in order to cover the usual range of concentrations of bioactive substances when investigated in vitro. Results obtained in this research allowed us to estimate the magnitude of global insult alterations caused by a drug (toxicity index) and the degree of participation of the different mechanisms of hepatotoxicity, acting simultaneously, at the cellular level.

## Materials and methods

### Standards and reagents

Methanol and acetonitrile (LCMS grade) were purchased from Sigma-Aldrich (Madrid, Spain), Dimethyl sulfoxide and formic acid were obtained from Sigma-Aldrich (Madrid, Spain). Ultra-pure water was obtained from a Milli-Q Integral Water Purification System from Merck Millipore (Darmstadt, Germany). Isotopically labelled standards phenylalanine-D_5_, tryptophan-D_5_ and caffeine-D_9_ were purchased from C/D/N Isotopes Inc (Quebec, Canada). All compounds used for incubations were obtained from Sigma-Aldrich (Madrid, Spain), and prepared as indicated (S. Table 1). The compounds of the training set are displayed in Table 1, properly grouped, accordingly to mechanisms.

**Table 1:** Compounds used in the training set along with their primary and secondary mechanisms of hepatotoxicity, according to scientific literature. Compounds used to investigate each mechanisms of hepatotoxicity are properly grouped, accordingly to mechanisms

Compound	IC10 (µM)	IC50 (µM)	Main mechanism	Second mechanism	Compound	IC10 (µM)	IC50 (µM)	Main mechanism	Second Mechanism
**Oxidative stress (OS)**					**Steatosis (ST)**				
Acetaminophen	500	2000	OS	APT	Acetylsalicylic	5000	20,000	ST	OS
Acetylsalicylic	5000	20,000	OS	ST	Levofloxacin	235	5800	ST	OS
Aflatoxin B1	100	500	OS	APT	Amiodarone	13	30	ST	MI
Amox–Clav (4:1)	1094/500	2190/1000	OS	CHOL	Tetracycline	100	2000	ST	MI
Azathioprine	173	600	OS	APT	Valproic acid	1000	5000	ST	MI
Chlorpromazine	0.5	60	OS	CHOL	**Apoptosis (APT)**				
Dantrolene	5	40	OS	NA	Acetaminophen	500	2000	APT	OS
Erythromycin	220	3200	OS	APT	Aflatoxin B1	100	500	APT	OS
Levofloxacin	235	5800	OS	ST	Azathioprine	173	600	APT	OS
Mercaptopurine	25	50	OS	APT	Captopril	250	500	APT	NA
Rifampicin	50	100	OS	NA	Erythromycin	220	3200	APT	OS
**Mitochondrial disruption (MI)**					Mercaptopurine	25	50	APT	OS
Amiodarone	13	30	MI	ST	Diclofenac	320	700	APT	MI
Bosentan	60	590	MI	CHOL	Troglitazone	50	300	APT	MI
Carbamazepine	200	1000	MI	NA	**Cholestasis (CHOL)**				
Clozapine	22	70	MI	NA	Amox-Clav (4:1)	1094/500	2190/1000	CHOL	OS
Diclofenac	320	700	MI	APT	Chlorpromazine	0.5	60	CHOL	OS
Flutamide	30	600	MI	NA	Bosentan	60	590	CHOL	MI
Isoniazid	1000	10,000	MI	NA	Thiabendazole	10	500	CHOL	NA
Phenytoin	100	500	MI	NA	**Non-Toxic**				
Stavudine	500	1000	MI	NA	DMSO	0.5	5	NT	NA
Tetracycline	100	2000	MI	ST	Glucose	5000	50,000	NT	NA
Troglitazone	50	300	MI	APT	Acetylcysteine	100	1000	NT	NA
Valproic acid	1000	5000	MI	ST	Thiamine	100	1000	NT	NA

Principal mechanisms of hepatotoxicity for each compound, oxidative stress (OS); mitochondrial disruption (MI); steatosis (ST); apoptosis (APT); cholestasis (CHOL); non toxic (NT); not assigned (NA), as attributed in the scientific literature

### Cell cultures, incubation with drugs and cytotoxicity assessment

HepG2 (ECACC No.85011430) cells were seeded and cultured to 70–80% confluence in 12-well plates, at a density of 300,000 cells/well, cultured with DMEM + 5% foetal calf serum, and kept in culture at 37 °C with 5% CO_2_/95% atmospheric air and saturated humidity for 24 h. Stock solutions for each chemical were prepared in dimethyl sulfoxide (DMSO) to a sufficiently high concentration so the final concentration of DMSO in culture media at the highest drug concentration assayed in cells was 1% (v/v). Each compound was added to culture media in quadruplicate wells and incubated for 24 h. Control culture wells were simultaneously incubated without adding any chemical. In addition, culture wells containing only media but no cells were included in the analyses as blank samples. For the determination of the IC10 and IC50 cytotoxic concentrations of chemicals, the MTT test (reduction of [3-4,5-dimethythiazol-2-yl]-2,5-diphenyltetrazolium bromide to a blue formazan) was used and concentration curves were graphically represented to determine the IC10 and IC50, by mathematical (logit curve) interpolation (Berridge and Tan 1993; Tolosa et al. 2015). Results are summarised in Table 1. An additional replicate culture plate was used for protein determination using Pierce™ Rapid Gold BCA Protein Assay Kit (by Thermo Scientific Pierce). Cell toxicity estimation and the contribution of each specific mechanism was first investigated in cells treated with a set of 25 compounds (25 hepatotoxic and 9 non-hepatotoxic, *training set*) and further validated with 87 compounds (69 hepatotoxic + 18 non-hepatotoxic) at 4 fixed concentrations (1, 10, 100, and 1000 µM), in quadruplicate wells for 24 h. Amiodarone, atorvastatin, azathioprine, cyclosporine a, fialuridine, tamoxifen and troglitazone compounds, due to their limited solubility, were incubated up to their highest concentration possible (see Supplementary Table 1).

### Sample preparation for metabolomics analysis

Consistent, broad and reproducible data of the cell's metabolites content is critical for meaningful readings of the metabolome. Thus, it is of critical importance a rapid cell quenching during sample collection to provide robust data for modelling. We optimised extraction of metabolites while following the recommendations of Dettmer et al. 2011 for mammalian cell harvesting, quenching and extraction (Dettmer et al. 2011), as well as other critical factors contributing to metabolomic data variability, such as cell pasage, sample peparation and storage, and equipment stability reading, as it was previously and extensively assessed by us (Martínez-Sena et al. 2019; Moreno-Torres et al. 2021). After incubation, culture media was removed and culture wells were rinsed twice with cold PBS. Immediately thereafter, cells were detached by scrapping whole plate twice using 300 µL of a solution of cold methanol:water (3:1 v/v) containing a set of internal standards (0.25 μM of phenylalanine-D5, tryptophan-D5 and caffeine-D9). The detached cells, suspended in quenching media, were collected and freezed and thawed three times in liquid nitrogen for cell disruption and metabolite extraction. Following centrifugation (10,000*g*, 10 min), supernatant was collected into a new Eppendorf tube, evaporated to dryness under vacuum, and reconstituted in 75 μL of 95:5 acetonitrile:water (0.1% formic acid) (v/v). A volume of 25 μL of each extract was withdrawn and pooled jointly to prepare a quality control (QC) sample for routine intra and inter day batch normalisation.

### Mass spectrometry-based metabolomic analysis

Metabolomic analysis was performed using an Agilent 1290 Infinity HPLC chromatography coupled to an iFunnel quadrupole time of flight (Q-TOF) Agilent 6550 spectrometer (Agilent Technologies, CA, USA). Samples were analysed using two different chromatographic conditions in order to separate and identify a sufficiently large number of metabolites. Method 1 used a Synergi Hydro-RP (150 × 1 mm, 4 µm Phenomenex, Torrance, USA) column at 50 °C, and injection volume was 3 μL. Binary mobile phase gradient starting at 99% of solvent A (water, 0.1% (v/v) formic acid) for 2 min, followed by a linear increase of solvent B (acetonitrile, 0.1% (v/v) formic acid) up to 80% in 8 min and rise to 98% in 0.1 min. Finally, 98% of solvent B was held for 2 min and then, initial conditions for 3 min to allow reconditioning of the column. The flow rate was set at 400 μL/min. Method 2 used a Waters Acquity UPLC BEH C18 (2.1 × 100 mm, 1.7 μm, Wexford, Ireland) column at a flow rate of 400 μL/min using the binary mobile phase system as in Method 1. Column was kept at 55 °C and the injection volume was 3 μL. Gradient elution was performed first with 98% of A, 2% of B held for 0.5 min, followed by a linear gradient of B from 2 to 20% in 4 min and from 20 to 95% B in 4 min. Finally, 95% B was held for 1 min and then, a 0.25 min gradient was used to return to the initial conditions, which were held for 2.8 min (Quintás et al. 2021). The chromatographic separation of highly polar metabolites was better achieved with procedure 1. Procedure 2, was more suitable for low polarity metabolites and successfully separated a wider range of analytes.

For the analysis, samples were randomly injected and acquisition of MS-data was open between 70 and 1200 m/z by positive electrospray mode, with the following parameters selected: gas temperature (T), 200 °C; drying gas, 14 L/min; nebulizer, 37 psig; sheath gas T, 350 °C; sheath gas flow, 11 L/min. To correct mass drifts during data acquisition, a set of mass reference standards (phthalic anhydride, purine, and hexakis (1H, 1H, 3H-tetrafluoropropoxy) phosphazene) were used. The QC sample, generated as described above, was injected every 8 injections for intra- and inter-batch correction.

### UPLC–MS data pre-processing and batch correction

Pre-processing of data for an untargeted analyte profile including peak detection, deconvolution, alignment and integration was performed by XCMS v 3.4.2 (Smith et al. 2006) software in R v 3.5.0. The centWave method was used for peak detection with the following parameters: mass accuracy = 20 ppm, peak width = (3, 12), snthresh = 12 and prefilter = (5, 5000). A minimum difference in *m*/*z* of 10 mDa was selected for overlapping peaks. Intensity weighted *m*/*z* values of each feature were calculated using the wMean function. Peak limits used for integration were found through descent on the Mexican hat filtered data. Matching peaks across samples was performed using the nearest method with mz-retention time (RT) balance of 2, RT tolerance of 3 s and kNN = 2. Missing data points were filled by reintegrating the raw data files in the regions of the missing peaks using the fillPeaks method.

For intra-batch correction, a non-parametric QC-SVRC approach was used as described elsewhere (Kuligowski et al. 2015). The batch effect between days was corrected by the ratio of a sample (QC of first batch), analysed repeatedly on each batch. LC–MS features with QC RSD > 30% after within batch effect correction were removed from analysis. Blank samples were employed to remove background and carry-over signals, features that were not at least three-fold higher than blank samples. All batches included QC, controls, non-toxic, toxic and blank samples and were randomly injected. The protocol for incubation, metabolite extraction and measurement had been previously explored to provide significant and reproducible cellular metabolite levels following compound treatment, (Moreno-Torres et al. 2021). Metabolomics data reproducibility was properly checked. Data obtained from several negative controls is displayed in Supplementary Fig. 1a; the relative standard deviation (RSD), mean and CI (95–5%) for six endogenous cell metabolites showed a mean RSD < 30%. A histogram of mean RSD distribution of all signals of four different batches is displayed and show that 92% of the signals have RSD < 30% on negative controls (Supplementary Fig. 1b). Additionally, (Supplementary Figure 1c) two compounds (amoxicillin–clavulanate and valproic acid) both present in the train and test sets at the same concentration (1000 µM) were compared. Peak area of four key metabolites (ophtalmic acid, hydroxybutyrylcarnitine, N-8 acetyl spermidine and prutrescine) are displayed along with the values recorded for the negative controls. It can be observed that the signals obtained maintain the same pattern trend (increase or decrease), notwithstanding the run, compared to control samples.

### Metabolite annotation

The fragmentation pattern of signals was extracted by MS/MS data-dependent acquisition using QC samples with the following *m*/*z* precursor ranges: 70–200, 200–350, 350–500, 500–650, 650–800, 800–950, 950–1100 and 1100–1200 Da. Features (*m*/*z*-RT) were annotated by matching experimental MS/MS spectra to MS/MS spectra available in the Human Metabolome Database (HMDB, www.hmdb.ca) corresponding to [M + H]^+^ precursors (*m*/*z* accuracy error < 20 ppm) as described elsewhere (Ten-Doménech et al. 2020). As a result, 116 features were successfully annotated (Supplementary Table 2), of which 67 and 49 metabolites were retrieved in Method 1 and Method 2, respectively.

### Bioinformatics data analysis and software

MS data were converted to mxZML and ms2 format using Proteowizard software (Chambers et al. 2012). Statistical analysis was carried out in MATLAB 2021a (Mathworks Inc., Natick, MA, USA) using in-house written scripts and the PLS_Toolbox 8.3 (Eigenvector Research Inc., Manson, WA, USA). Multivariate analysis was carried out by Principal Component Analysis (PCA) and Partial Least Squares-Discriminant Analysis (PLS-DA). PLS-DA allowed us, first to identify the most relevant biomarkers for each mechanism of toxicity and second, to mechanistically classify compounds based on the induced changes of the metabolic profile (Worley and Powers 2013). Threefold cross validation (CV) (Refaeilzadeh et al. 2016) was perfomed to estimate the out-of-sample PLS–DA prediction error. The number of PLS latent variables (LVs) was selected according to the lowest error of classification. The optimal threshold was determined by the intersection of sensitivity and specificity curves (Pérez et al. 2009). The assessment of the statistical significance of PLS figures of merit was carried out by permutation testing. The Pathway Analysis module on Metaboanalyst 5.0 website was applied using the metabolite peak intensities as input data (Pang et al. 2021). Metabolic pathway data were matched against the human KEGG database (Kanehisa et al. 2021). SVR models estimated for within-batch effect elimination were carried out in MATLAB using the LIBSVM library (Chang and Lin 2011).

## Results and discussion

### Strategy overview

To improve the metabolite annotation, samples were analysed using two complementary methods. According to their distinct physicochemical properties, the chromatographic separation of highly polar metabolites was best achieved in the Synergi Hydro RP (Method 1); while metabolites with lower polarity showed a better chromatographic separation in the Waters Acquity column (Method 2).

The data set of metabolites identified from both chromatographic methods was combined. Data retrieved using method 1 and method 2 included 67 and 49 identified metabolites, respectively.

**Fig. 1:**
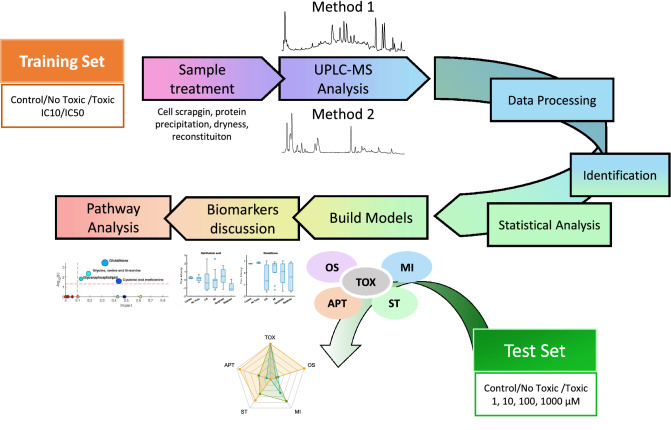
Workflow diagram of the research undertaken. Using a training set of compounds at IC10/IC50 concentrations, metabolomic data were retrieved by two analytical procedures and predictive models of toxicity (TOX) and hepatotoxicity mechanisms (OS, MI, APT, ST) were subsequently built. The biomarkers identified so far, for global toxicity and for each toxicity mechanism were discussed and examined in the context of metabolic pathway analysis. A test set of 87 compounds containing hepatotoxic compounds acting through different mechanisms, as well non-hepatotoxic compounds was subsequently evaluated and results were displayed in a radar chart, in order to account for the participation of the different mechanisms of toxicity and the outcome of this informtion discussed and compared to the occurrence of mechanisms previously reported in the literature

The workflow of the research undertaken, its rationale and the overall strategy applied is shown in Fig. 1. In the study, a *training set* of compounds containing drugs representative of the different mechanisms of hepatotoxicity were used. Upon cell incubation at two different concentrations, cell extracts were analysed by UPLC–MS to retrieve a broad range of signals that enabled the identification of metabolic patterns associated with specific mechanisms of drug-induced hepatotoxicity, different from those of general cytototoxicity.

For model generation, we used the metabolome of cells treated with compounds predominantly acting via one mechanism of hepatotoxicity, and compared with the metabolome of cells treated with the rest of the compounds, starting at the lower concentration, and further comparing with the metabolome of cells treated with non-toxic compounds and non-treated control cells. At the IC50 concentration, only alive and attached cells metabolomes were analyzed and both types of signals, those related to general toxicity as well those linked to the occurence of a specific mechanism of hepatotoxicity were recorded.

Following this, biomarkers emerging as specific for global toxicity and for individual mechanisms of toxicity were properly identified. Metabolic pathway analysis was also conducted to confirm the relevance of each metabolite to the corresponding mechanism of toxicity. We then built predictive model based on these biomarkers relevant for global hepatotoxicity and for each of the individual mechanisms of hepatotoxicity. Following this, the developed models were further validated by examining the metabolomic pattern of toxicity of cells incubated with a larger set (87) of test compounds.

### Global drug-induced hepatotoxicity model

A prediction model for assessing global hepatotoxicity (TOX) was constructed. Autoscaled metabolic profiles from cells exposed to two toxic concentrations IC10 and IC50 (Table 1) were selected and compared with profiles obtained from cells exposed to equivalent concentrations of non-hepatotoxic compounds, as well non-treated cells (controls). These two concentrations were selected to produce a broad range of signals for mechanism modelling. For the model generation, we used the metabolome of cells treated with compounds predominantly acting via one mechanism of toxicity, starting at the lower concentration (IC10), and compared with the metabolome of cells treated with non-toxic compounds and non-treated control cells. The IC50 concentration, as determind by the MTT test, is a concentration that affects 50% of the mitochondrial activity, while cell viability is much less affected. The metabolome obtained from cells treated at high concentrations (IC50) may contain signals attributable to general cytotoxicity events, overlapping, but not abolishing, the signals linked to a specific mechanism of drug-induced hepatotoxicity. Therefore, at this concentration we are recording both mechanism-related signals and general toxicity signals as well. Later, by doing these comparisons for each mechanism, at high and low concentration, we were able to distinguish the metabolites specifically altered by each toxicity mechanism from the metabolites arising in general toxicity, irrespective of the drug used. Hence, this strategy enabled us to discriminate relevant metabolomic changes linked to each mechanism of toxicity, from those less specific arising from a general cytotoxicity event (see below).

**Fig. 2:**
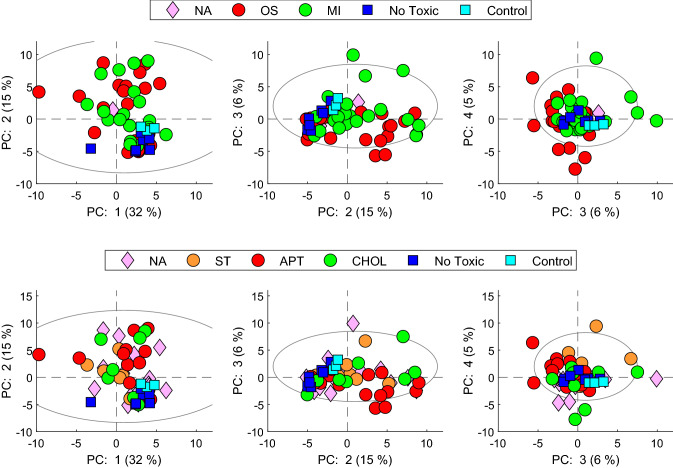
Principal component analysis on training set. PCA scores plots of the combined metabolome from cells treated with IC10 and IC50 concentrations of hepatotoxins acting through different mechanisms (oxidative stress (OS); mitochondrial disruption (MI); no toxic, (upper row); steatosis (ST); apoptosis (APT); cholestasis (CHOL), non toxic (NT), lower row; as well as with non-toxic xenobiotics, not assigned (NA) and controls. There is a clear separation trend among toxic and non-toxic concentrations/compounds

As a first approximation, we performed an unsupervised Principal Component Analysis (PCA) of the autoscaled data signals of identified metabolites from cells treated with drugs at IC10 and IC50. The four first principal components explained approximately 60% of the data variance. For better visualisation, six PCAs are shown in Fig. 2, showing the main (MI, OS) and secondary (APT, ST, CHOL) mechanisms of toxicity. In the PCA scores, only a slight separation of the samples according to the different mechanism of hepatotoxicity was observed when analysing the whole set of data. The analysis of the spatial distribution of objects in a PCA scores space is widely used to identify the main sources of variation and to get a first overview of the data structure and to identify potential outliers. However, this is an unsupervised approach and the interpretation of the sources of variation might be difficult, if they are the result of a combination of biological effects associated with one or more (orthogonal) principal components. Besides, other elements, like the technical and instrumental effects, might also have an impact (indeed, they can be one of the main sources of variance). Thus, even if in a PCA score plot there is no clear sample clustering associated with a given intervention (e.g. toxicity mechanism), it cannot be ruled out that there might be an impact on the metabolomic profile. Notwithstanding, what can be observed is that there is a clear separation trend among toxic and non-toxic concentrations/compounds.

**Fig. 3:**
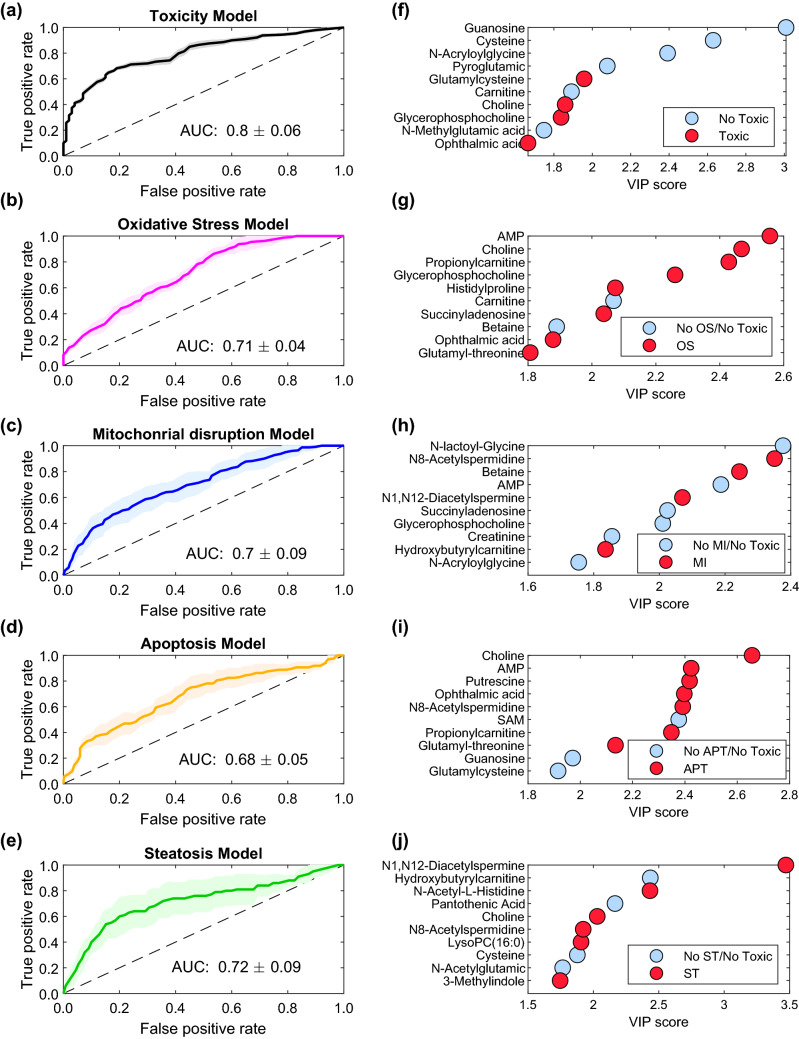
ROC curves for global and individual toxicity mechanisms prediction models. The average ROC curves for TOX (**a**), OS (**b**), MI (**c**), APT (**d**) and ST (**e**), with standard deviation (std) on shadow, as well the AUC value (mean ± std). Top VIP scores for the corresponding prediction model (**f**–**j**). Red circles indicate higher metabolite concentrations in the corresponding toxic mechanism. On the contrary, blue circles refer to metabolites displaying high concentration in the negative mechanism group VIP scores of each model for all metabolites are displayed on Supplementary Table 2

This first approach was followed by a partial least squares-discriminant analysis (PLS-DA) performed with data of the identified metabolites from cells incubated with toxic compounds at IC10 and IC50 *vs* non-toxic and control samples. In Table 2, we can observe the distribution of the samples in the different classes of the prediction models. We carried out a repeated (*n* = 10) random threefold CV (Lee et al. 2018) and 6 LVs were selected according to the lowest value of error rate (Supplementary Fig. 2a). Figure 3a shows the receiver operating characteristic curve (ROC) estimated by CV. The value of the area under curve (AUC) was 0.8, indicating a high performance for toxicity classification and a good sensitivity and specificity in discriminating hepatotoxic compounds from controls and non-toxic compounds. The statistical significance of the PLS-DA model was assessed by permutation testing (200 permutations, *p *value < 0.05) as described elsewhere (Neubert and Brunner 2007), using the AUROC as target function.

**Table 2:** Balance of positive and negative probes in the training set for each class and mode of action. Oxidative stress (OS); mitochondrial disruption (MI); steatosis (ST); apoptosis (APT); and cholestasis (CHOL)

Model						
	TOX	OS	MI	APT	ST	CHOL
Positive probes	50	24	22	16	10	8
Negative probes	10	36	38	44	50	52

### Predictive models for the different mechanisms of drug-induced hepatotoxicity

Then, a set of classification models to enable the discrimination among toxicity mechanisms (OS, MI, APT, ST and CHOL) was built using the metabolic profiles of cells incubated with the appropriate model compounds (Table 1). Accordingly, for each of the five considered mechanisms of toxicity a *one vs all* discriminant model was built, in which samples obtained from cells incubated with compounds inducing a given type of toxicity were compared to samples obtained from cells incubated with either non-toxic or compounds inducing a different type of toxicity.

We made a comparison of metabolomes from cells treated with low concentrations of toxic compounds at IC10 concentration, non-toxic compounds and non-treated cells. This was complemented with data obtained at higher concentrations (IC50) which enabled us to filter off signals attributable to general toxicity while emerging the mechanism-specific metabolome signals. In this way, the analysis for each mechanism allowed us to identify metabolites altered specifically by each toxicity mechanism and to exclude effects linked to general toxicity. Based on these metabolite changes, metabolic pathways analysis was also performed for each mechanism of toxicity and subsequently analysed and compared among them to confirm their relevance for a specific mechanism of toxicity.

Then, we built a predictive model based on the biomarkers relevant for global hepatotoxicity and those relevant for each of the individual mechanisms of hepatotoxicity (i.e., comparing the metabolome of cells incubated with compounds that exerted its action through a given mechanism with the rest of compounds). Model performance and statistical significance was carried out as for the Global drug-induced hepatotoxicity model. The number of LVs selected for each model is shown in Supplementary Fig. 2b–f. Supplementary Fig. 3 depicts the evolution of the sensitivity and selectivity of each PLS-DA model as a function of the discrimination threshold employed. Based on these results, the optimal classification thresholds for each model were 0.63, 0.35, 0.35, 0.28 and 0.19 for TOX, OS, MI, APT and ST, respectively (Supplementary Fig. 3). Figure 3a–e shows ROC curves for global toxicity model (a) and for each mechanism model (b–e) showing AUC mean values between 0.68 and 0.81, supporting the discrimination performance of the models. Nonetheless, the model built for the identification of CHOL toxic responses showed insufficient sensitivity and specificity (AUC < 0.5) (Supplementary Fig. 4). A possible explanation is that whereas HepG2 cells perform most of the mature hepatocyte metabolic functions, they have very limited ability for bile acid synthesis (Everson and Polokoff 1986) and have a reduced expression of the principal bile acids transporters (Kullak-Ublick et al. 1996), thereby limiting their applicability as an in vitro model for the detection of metabolic alterations caused by cholestatic drugs (Cooper et al. 1994).

A slightly lower predictive performance of the individual mechanistic models compared to the global hepatotoxicity model was observed. This could be attributed to the fact that there are no toxic compounds acting exclusively through a unique toxic mechanism, but to an interrelation of them within the cell which depends on the exposure conditions (i.e., concentration, time). Given this unavoidable overlapping effect of distinct mechanisms, it is understandable that the compounds, although classified as representative of one given mechanism of hepatotoxicity, may partially display biomarkers associated with other mechanisms of hepatotoxicity.

**Fig. 4:**
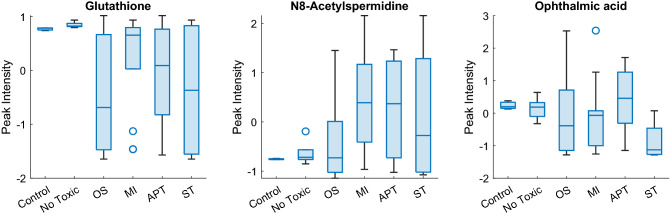
Boxplots of autoscaled peak intensities from a set of metabolites and their relevance in the mechanisms of hepatotoxicity. Toxic samples were grouped by mechanism as oxidative stress (OS), mitochondrial disruption (MI), Apoptosis (APT) and steatosis (ST). These biomarkers, typically absent in non-toxic compounds or controls, are equally recognised in the literature as biomarkers of the different types of hepatocyte damage. Autoscaling of each variable was carried out by mean centering followed by dividing by the standard deviation

The most relevant metabolites for global hepatotoxicity assessment and for each mechanism prediction model (VIP > 1.5) are shown in Fig. 3f–j. Information about the identified metabolites is summarised in Supplementary Table 2 including *m*/*z*, retention time, HMDB code, metabolic pathway, VIP scores, and* t* test *p* value of each prediction model. Three relevant identified metabolites with VIP > 1 are grouped by type of mechanism and displayed in Fig. 4. Decreased levels of Glutathione (GSH) emerged as a consistent biomarker of oxidative stress mechanisms. GSH is involved in conjugation processes being of great relevance in liver detoxification processes (Kaplowitz 1981; Irie et al. 2016). Polyamines and their acetylated products N1-acetylspermine and N8-acetylspermidine showed high VIP scores in the MI, APT and ST models, and low VIP scores in the OS model. It is known that polyamines play an important role in the regulation of mitochondrial Ca^+2^ transport and ATP (Salvi and Toninello 2004) and are related to an increase of oxidative stress independent of the glutathione pathway (Rider et al. 2007). Moreover, the spermidine/spermine N (1)-acetyltransferase (SSAT) enzyme can be induced in the liver by toxins such as carbon tetrachloride (Matsui et al. 1981) resulting in an increase of N1-acetylated polyamines as well as SSAT is reported to be located mainly in mitochondria (Holst et al. 2008). Furthermore, in agreement with previous results ophthalmic acid was found highly significant (VIP > 1.5) in the OS and APT models. Besides, the concentration profile in OS is associated with GSH depletion observed in the course of oxidative stress (Soga et al. 2006). Ophtalmic acid resulted more relevant to OS, MI and ST, and differ from non toxic compounds.

### Metabolic pathway analysis

**Fig. 5:**
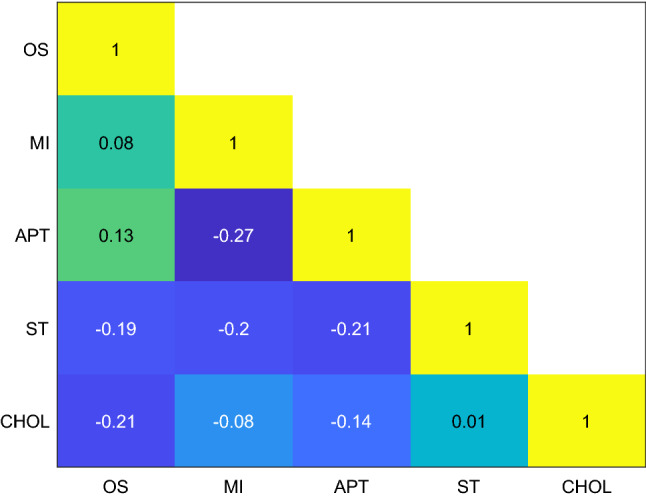
Correlation between paired comparisons of pathway analysis evaluated using the Mantel’s test for the different mechanisms assessed. Changes in pathway analyses were inferred after comparing samples treated with drugs acting through a given mechanism of toxicity versus control samples. No significant correlation (permutation test *p* value < 0.05) was found among the results from metabolic pathway analysis observed for the different mechanisms. This indicates that impacts in the metabolic pathways are different for each mechanism of toxicity

The identification of the alterations in metabolic pathways associated with the different toxicity mechanisms was carried out by Metabolic Pathway Analysis (MetPA). For that purpose, MetPA was performed comparing samples from each mechanism versus control samples. MetPA combines several advanced pathway enrichment analysis procedures along with the analysis of pathway topological characteristics to help identify the most relevant metabolic pathways involved in a given metabolomic study (Xia and Wishart 2010). A list of all metabolites included and their corresponding HMDB ID are shown in Supplementary Table 2. Results from pathway analysis were summarised with 2 descriptors (− log10 (*p* value) and the impact factor) as described elsewhere (Xia and Wishart 2010), using metabolic pathways with > 3 hits for the analysis. Different metabolic pathways appeared significantly altered within each mechanism (Supplementary Fig. 5) when compared to controls. To determine whether these altered pathways were specific and could constitute a characteristic metabolic fingerprint of each mechanism of hepatotoxicity, a correlation analysis was performed to compare among the metabolic pathways altered in cells treated with drugs acting by one of the mechanisms with respect to controls cells, versus the metabolic pathways altered in cells treated with drugs characteristic of other mechanisms with respect to controls cells. The pairwise correlations between MetPA outcomes obtained for APT, ST, OS and MI were estimated using Mantel's test, as previously described (Ten-Doménech et al. 2021). In this study, the −log10(*p* value), and the impact factor (estimated as the sum of the importance of the measures of all metabolites in the pathway) were used as descriptors (i.e., coordinates) of the MetPA outcomes. Finally, the Euclidean distance between metabolic pathways was used as a measurement of dissimilarity between metabolic pathways. For a better understanding, this strategy is highlighted in the workflow shown in Supplementary Fig. 6. The correlation between dissimilarity vectors was estimated using the Pearson correlation as we previously described in detail (Moreno-Torres et al. 2021; Ten-Doménech et al. 2021). This test estimates a correlation score between the outcomes of two MetPA (Z_M_). A high, statistically significant score indicates a strong correlation between the two distance matrices containing the pairwise distances between the elements of each set, where small and large distances in one MetPA are associated with small and large distances in the second MetPA. Supplementary Table 2 shows the metabolites involved in each pathway. As described above, glutathione and other metabolites of the glutathione pathway were affected in the course of hepatotoxicity, being relevant to all mechanisms of toxicity. The nicotinate and nicotinamide pathway were altered only in the ST mechanism. Indeed, it has been already described in the literature that a decrease in the coenzyme NAD, characteristic of this pathway, is related to an increase in ST (Mukherjee et al. 2017) and we found this metabolite with VIP > 1 in the ST model. Results also agree with the literature in the sense that the glycerophospholipid pathway is altered in the course of MI and ST (Peng et al. 2018). Drugs inducing cholestasis did not significantly influence any metabolic pathway with the currently annotated metabolites. as previously mentioned. This is likely due to the fact that HepG2 cells lack the ability to synthesise, conjugate and transport bile acids (Everson and Polokoff 1986). Results depicted in Fig. 5 describe the correlation coefficient between paired comparisons of pathway analysis of the different mechanisms assessed and their statistical significance evaluated by the Mantel’s test. They show low, non-statistically significant correlations among the alterations observed for the different mechanisms. This result suggests that, despite some metabolic pathways are commonly altered across mechanisms, the overall impact on the metabolome remains characteristic of each toxicity mechanism. Thus, by evaluating the meta-analysis of results from the pathway analysis, a specific metabolic fingerprint for each toxicity mechanism could be obtained.

### Assessment of the global toxicity and toxicity mechanisms prediction models on a set of testing compounds

The performance of *global toxicity and toxicity mechanisms* models to identify the contribution of each mechanism to the global hepatotoxicity were assessed in a test set of 87 compounds having been identified in the literature as acting via a principal mechanism of hepatotoxicity. Compounds were examined at a range of increasing concentrations (from 1 to 1000 μM), as an unknown compound would have been assayed in a blind in vitro test (Gómez-Lechón et al. 2010; Tolosa et al. 2012b; Chen et al. 2016), to see whether the model could reveal the occurrence of general toxicity and/or the contribution of the various mechanisms of toxicity at increasing concentrations (Concentrations assayed are reported in Supplementary Table 1; in some cases, the largest assayable concentration, because of compound's solubility is indicated).

PLS y-predicted values accounted for the magnitude of global toxicity, as well for the relative impact of each of the mechanisms of hepatotoxicity (OS, MI, APT and ST) associated with each compound at each assayed concentration. Low protein content in cell homogenates (less than 1/3 of that observed in blank samples) was a sample exclusion criterion, as it was indicative of extensive cell death that might distort the intracellular metabolome. Thus, these samples were excluded from the validation set (see the indication of low protein (LP)). Values for all compounds and all concentrations are summarised in Supplementary Table 3 together with the mechanism of toxicity attributed in the literature to each compound (Gómez-Lechón et al. 2010; Tolosa et al. 2012b). In Supplementary Fig. 7 the predicted toxicity index values of toxic compounds at the different concentration ranges (i.e., 0–1 µM; 1–10 µM; 10–100 µM, > 100 µM) is displayed. All compounds were examined at a wide range of concentrations, to assess whether the model identifies a general hepatotoxicity and/or the contribution of the various mechanisms of toxicity at each concentration assayed. Application of the model to each compound and each concentration revealed that a given mechanism of hepatotoxicity could predominate at a certain concentration, but at higher concentrations other mechanisms of toxicity would also be present and contribute to the compound’s global toxicity. Results showed, as expected, an upward trend in the toxicity index with increasing doses, irrespective of the toxicity mechanisms elicited by the drug.

As observed in the Supplementary Fig. 8, 80% of compounds named in the scientific literature as hepatotoxins were correctly classified as toxic when incubated at 1000 µM (or maximum concentration). It is also remarkable that the MI mechanism appears at lower concentrations as opposed to the other mechanisms where its incidence increases with concentration. MI is likely to appear in the early stages of toxicity mechanisms. Despite some disparity with the bibliography, each compound is classified within the mechanisms predicted in at least one of the concentrations used. It is remarkable to appreciate that our model predicts the occurrence of overlapping of mechanisms in more than 90% of the studied compounds. This seems to be the expected situation where two or more mechanisms occur at a time or sequentially.

Radar chart was used to integrate the outcomes from the analysis of the five models (TOX, OS, MI, APT, ST) and to visualise the participation of each of them in the validation compounds set (87), and to easily interpret the metabolic changes observed along the four concentrations tested. The participation of each mechanism of toxicity is represented in the radar chart; the different axes graphically represent the y-predicted values from each mechanistic model, in a relative scale, ranging from 0 (no participation) to 1 (full participation). Combined prediction plots of 8 representative hepatotoxic compounds, acting preferentially via a specific mechanism (Gómez-Lechón et al. 2010): (a) OS, (b) MI, (c) APT and (d) ST, are displayed in Supplementary Fig. 9. Results showed that although claimed in the literature to act *preferentially* via a given mechanism, such mechanism is not unique, and others are likely to be involved. Indeed, while the global toxicity index generally increased with concentration, the relative contribution of each toxicity mechanism to global hepatotoxicity was influenced by the concentration being assayed. Thus, although cumene hydroperoxide and Mercury II are claimed to cause OS (Gómez-Lechón et al. 2010; Tolosa et al. 2012b), and indeed this is the case according to metabolome analysis, other metabolic alterations (APT, ST and MI) are present at larger concentrations in cumene hydroperoxide, but not in Mercury II (Supplementary Fig. 9a). 2,4-Dinitrophenol and azathioprine (Supplementary Fig. 9b) are regarded as causing MI (Gómez-Lechón et al. 2010; Tolosa et al. 2012b). But this is more evident in 2,4 dinitrophenol at all concentrations, while in the case of azathioprine is more evident at lower concentrations, being overridden by OS, APT and ST at the highest concentration, in agreement with the bibliography. Aflatoxin B1 and etoposide are claimed as eliciting APT in hepatocytes (Gómez-Lechón et al. 2010; Tolosa et al. 2012b). This is indeed observed at higher concentrations in the case of aflatoxin B1, but it is less evident in the case of etoposide, where other mechanisms (OS) prevail over APT, although they are indubitably related (Supplementary Fig. 9c). Chlorpromazine and imipramine, claimed to cause ST (Gómez-Lechón et al. 2010; Tolosa et al. 2012b), do alter hepatocyte metabolome in this sense, at a high concentration, but other mechanisms are involved as well (Supplementary Fig. 9d).

**Fig. 6:**
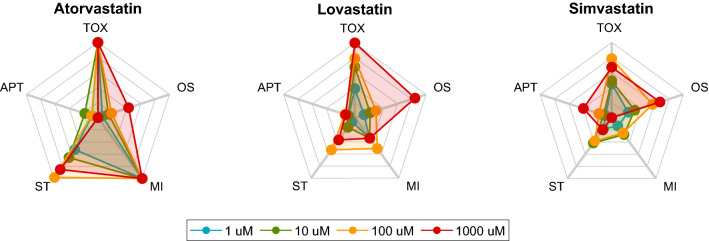
Integrative toxicity radar chart for 3 representative statins. The metabolome of cells incubated with three statins, described in the literature as causing MI (Atorvastatin), OS (Lovastatin) and APT (Simvastatin) as principal mechanisms of hepatotoxicity were examined at various concentrations. The global toxicity and the participation of the different mechanisms of hepatotoxicity were estimated accordingly with the models and represented using integrative radar charts. Contribution of the different mechanisms of hepatotoxicity at various concentrations is displayed. The participation of more than one mechanism is evidenced and a different degree of mechanistic contribution to toxicity is observed with increasing concentrations

To better illustrate the utility of the developed tools, we applied the models to three members of the same class of drugs, statins, potent hypocholesterolemic drugs, and compared their effects on the metabolome of HepG2 cells when assayed at 4 different concentrations (Fig. 6). Both atorvastatin and lovastatin showed, as mentioned in the literature, a mechanistic pattern of MI and OS, respectively (Gómez-Lechón et al. 2010). However, simvastatin, often described as apoptotic in the literature, shows a predominant OS pattern at the highest concentration. This apparent inconsistency can be explained, as suggested by other researchers, by the fact that the APT in simvastatin is preceded by an OS phenomenon (Qi et al. 2010).

In Supplementary Fig. 10, we have included 4 compounds that show remarkable changes in the mechanism of hepatotoxicity with concentration. Fluoxetine and Clozapine are named in the literature as causing OS; Troglitazone as MI and APT and Rifampicin as OS and MI (Gómez-Lechón et al. 2010; Tolosa et al. 2012b). In the case of Fluoxetine, the described mechanism of OS is correctly observed at concentrations of 100 and 1000 µM. On the contrary, Clozapine, also described as OS in the scientific literature, shows evolution from an MI mechanism at the lowest concentrations to an OS mechanism. Troglitazone at lower concentrations main mechanism was MI and is taken over by OS at higher concentrations as described in the bibliography (Smith 2003). Meanwhile, Rifampicin coincides with the literature description as causing MI at lower concentrations, while the mechanism that predominates at higher concentrations is OS (Chowdhury et al. 2006).

## Conclusions

We have explored the analytical capacity of UPLC–MS/MS based metabolomics to consistently detect, quantify and identify changes in the metabolome of HepG2 cells metabolome when incubated with a set of hepatotoxic and non-hepatotoxic compounds and, based on this, to build predictive models to estimate the overall hepatotoxicity insult and the involvement of the different mechanisms of hepatotoxicity. Based on the occurrence of such metabolomic profiles we constructed predictive models to account for the likeliness of a compound to be hepatotoxic and to identify the hepatotoxicity mechanisms so far involved. Moreover, identification of key altered metabolites for each toxicity mechanism and the application of predictive models, enabled us to estimate the degree of participation of each of the mechanisms in the overall toxicity caused by a compound at each concentration.

An analysis of the different altered pathways is a powerful tool to draw conclusions on the metabolic changes caused in the cell under different conditions (Chen et al. 2015; Moreno-Torres et al. 2022). Cholestasis biomarkers in vitro could not be properly identified in this exercise, most likely because of the inability of the HepG2 model cell system to synthesise bile acids and to uptake and transport bile acid conjugates. Even if an oxidative stress mechanism (OS) is the key initiating event, a generalised OS damage will also cause changes in mitochondrial function and is related to highly prevalent diseases in the population such as Nonalcoholic fatty liver disease (NAFLD), steatosis and cirrhosis (Cichoż-Lach and Michalak 2014). In a similar manner an initial disruption of the mitochondrial function (i.e., mitochondrial membrane potential) will certainly cause broader changes as oxidative stress (Chowdhury et al. 2006), affecting other mitochondrial functions. Thus, the toxic effects of a given drug may result in a set of metabolic changes which are shared by more than one mechanism, and that we interpret as evidence of the involvement of more than one toxic pathway at this is something that can be observed in several of the molecules studied, where the contribution of other mechanisms to global hepatotoxicity may appear or evolve with increasing concentrations of the compound, as it has been already reported for some drugs (i.e. sorafenib (Rodríguez-Hernández et al. 2020), or the well-known discrepancies reported for the mechanism of toxicity of statins in the literature (Gómez-Lechón et al. 2010; Qi et al. 2010).

This study was designed as a *proof-of-concept* study, and we made use of a sufficient large number of hepatotoxic compounds acting principally, but not exclusively, via a major toxicity mechanism. Hence, this study has certain limitations regarding the experimental design. The fact that most of the compounds analysed in this study may act marginally through several mechanisms of drug-induced hepatotoxicity at increasing concentrations, makes more difficult the development of fully discriminant models for the identification of individual mechanisms of toxicity. Yet, the results obtained are eye catching and intuitively describe the hepatotoxic behaviour of compounds at different concentrations and the contributions of the different mechanisms of tocixity.

In summary, we have developed an innovative and efficient tool for the analysis and prediction of hepatotoxicity of drugs based on the metabolomic analysis of HepG2 cells exposed to several and different compounds. These models provide information on the overall hepatic alterations, metabolic pathways altered, as well the contributions of the mechanisms of hepatotoxicity so far involved. 

## Supplementary Information

Below is the link to the electronic supplementary material.

**Supplementary Fig. 1.** Figures illustrate relative standard deviation (RSD) of metabolomic data of negative controls in the different batches. a) Mean RSD and CI (95-5%) of six major cell metabolites peak areas (showing RSD < 30%. b) Histogram of the mean RSD of four different batches that shows that 92% of signals of negative controls have a RSD<30%. c) Peak area of four key metabolites displayed for two different negative controls and two hepatotoxic compounds (valproic acid and amoxicillin:clavulanic acid). **Supplementary Fig. 2.** Selection of the optimum number of latent variables. The graph depicts the progression of the Root Mean Square Error of Cross Validation (RMSECV) values obtained by the partial least squares (PLS) models with latent variable (LV) increments for the TOX (a), OS (b), MI (c), APT (d), ST (e) and CHOL (f) prediction models. **Supplementary Fig. 3.** Decision plot of prediction models for TOX (a), OS (b), MI (c), APT (d), ST (e) and CHOL (f) models to identify optimal decision threshold (Threshold selection for each model which achieves highest sensitivity and specificity). Below the calculated threshold, the sample cannot be classified as positive. **Supplementary Fig. 4.** Curves of cholestasis prediction model. Left: ROC curve (average) for CHOL with standard deviation displayed (shadowed), as well the AUC value (mean ± sd). Right: Top VIP scores above 1.5 in the CHOL model. **Supplementary Fig. 5.** Pathway Analysis of  metabolic changes associated to the different mechanisms of hepatotoxicity. Displayed are the altered pathways associated with various mechanisms of hepatotoxicity studied, a) OS; b) MI; c) APT; d) ST. Only pathways with a p-value <0.05 and impact>0.01 were considered. **Supplementary Fig. 6.** Schematic workflow for the metabolic pathway analysis. A t-test is developed to each mechanism (A and B) versus control samples with the metabolites identified and known pathway. Results from each pathway analysis are summarised with two descriptors (the -log10 (p-value) and impact factor) as coordinates of two data matrices. Then, the lower triangular part of each dissimilarity matrix is unfolded into two vectors to calculate the pairwise linear correlation coefficient between them. The statistical significance of the calculated correlation coefficient between the pathway analysis is estimated using a permutation test and the correlation coefficient is computed for each permutation. **Supplementary Fig. 7.** Prediction of global toxicity for 86 compounds assayed at 1, 10, 100 and 1000 µM. Distribution of Y-predicted values (toxicity index) calculated by the PLS globlal toxicity model, sorted by concentration. The dashed vertical lines delimit the concentration of the compounds being assayed. **Supplementary Fig. 8.** Summary of the classification of tested compounds according to mechanisms, at the different concentrations. Percentage of coincidence between the positive classifications of the predicted mechanism with regard to that of the literature at every concentration tested. **Supplementary Fig. 9.** Integrative Radar Chart displaying the participation of the different mechanisms in the global hepatotoxicity elicited by selected compounds at different concentrations. Radar charts display the degree of involvement of the different mechanisms of hepatotoxicity of hepatotoxic compounds claimed in the literature to act preferentially act via: OS (cumene hydroperoxide and mercury II); MI (2,4-dinitrophenol and azathioprine); APT (aflatoxin B1 and etoposide); and ST (chlorpromazine and imipramine), accordingly to the literature. **Supplementary Fig. 10.** Compounds fluoxetine (OS), chozapine (MI, OS), troglitazone (MI, AP) and rifampicin (OS, MI) show a changing pattern of hepatotoxicity mechanism involved with increasing exposure concentration.

**Supplementary Table 1.** List compounds of the test set. Compounds assayed are shown along with their CAS number. Four concentrations were investigated 1.10, 100 and the maximum indicated in the table (conditioned by the solubility in DMSO), and the stock solvent used. **Supplementary Table 2.** List of identified metabolites by untargeted metabolomics. All identified metabolites were shown including mass-to-charge ratio (m/z), retention time (RT), type of column (Synergy for Method 1, Acquity C18 for Method 2), HMDB code, metabolic pathway,VIP scores, and t-test p-value of each prediction model (TOX, OS, MI, APT, ST). VIP scores higher than 1 are shown as red or blue on whether its weight on the model is higher in the samples classified as mechanism positive or negative, respectively. p-value < 0.05 is statistically significant and coloured as green. **Supplementary Table 3.** Summary of the toxicity indexes for global and specific mechanism of toxicity for the test set of compounds analysed at each concentration, and the mechanisms described in the literature.

## Data Availability

The dataset generated during the current study is available on the Zenodo repository under 10.5281/zenodo.6411855.

## Abbreviations

APT: Apoptosis
AUC: Area under curve
CHOL: Cholestasis
CV: Cross validation
IC: Inhibitory concentration
LV: Latent variable
MI: Mitochondrial disruption
PCA: Principal component analysis
PLS-DA: Partial least squares-discriminant analysis
ROC: Receiver operating characteristic curve
RMSECV: Root mean square error of cross validation
ST: Steatosis
VIP: Variable importance projection
OS: Oxidative stress
TOX: Toxicity
