# Supplementary material for: Metabolomics-based strategy to assess drug hepatotoxicity and uncover the mechanisms of hepatotoxicity involved
Source: Arch Toxicol. 2023 Apr 6;97(6):1723–38. doi: 10.1007/s00204-023-03474-8 (PMC10182947; doi:10.1007/s00204-023-03474-8)
Supplement: Supplementary file 1 — Supplementary Fig. 1. Figures illustrate relative standard deviation (RSD) of metabolomic data of negative controls in the different batches. a) Mean RSD and CI (95-5%) of six major cell metabolites peak areas (showing RSD < 30%. b) Histogram of the mean RSD of four different batches that shows that 92% of signals of negative controls have a RSD<30%. c) Peak area of four key metabolites displayed for two different negative controls and two hepatotoxic compounds (valproic acid and amoxicillin:clavulanic acid). Supplementary Fig. 2. Selection of the optimum number of latent variables. The graph depicts the progression of the Root Mean Square Error of Cross Validation (RMSECV) values obtained by the partial least squares (PLS) models with latent variable (LV) increments for the TOX (a), OS (b), MI (c), APT (d), ST (e) and CHOL (f) prediction models. Supplementary Fig. 3. Decision plot of prediction models for TOX (a), OS (b), MI (c), APT (d), ST (e) and CHOL (f) models to identify optimal decision threshold (Threshold selection for each model which achieves highest sensitivity and specificity). Below the calculated threshold, the sample cannot be classified as positive. Supplementary Fig. 4. Curves of cholestasis prediction model. Left: ROC curve (average) for CHOL with standard deviation displayed (shadowed), as well the AUC value (mean ± sd). Right: Top VIP scores above 1.5 in the CHOL model. Supplementary Fig. 5. Pathway Analysis of metabolic changes associated to the different mechanisms of hepatotoxicity. Displayed are the altered pathways associated with various mechanisms of hepatotoxicity studied, a) OS; b) MI; c) APT; d) ST. Only pathways with a p-value <0.05 and impact>0.01 were considered. Supplementary Fig. 6. Schematic workflow for the metabolic pathway analysis. A t-test is developed to each mechanism (A and B) versus control samples with the metabolites identified and known pathway. Results from each pathway analysis are summarised with two descriptors ( [file 204_2023_3474_MOESM1_ESM.pdf]

a)

Intraday negative controls RSD (%)			
	RSD mean	RSD 95% CI	RSD 5% CI
Spermidine	13.7	16.9	11.9
L-Tryptophan	13.6	21.1	8.2
Methionine	12.1	18.6	3.8
GSH	7.2	11.7	2.8
Butyril Carnitine	10.0	14.8	7.8
Choline	27.2	32.0	23.3

b)

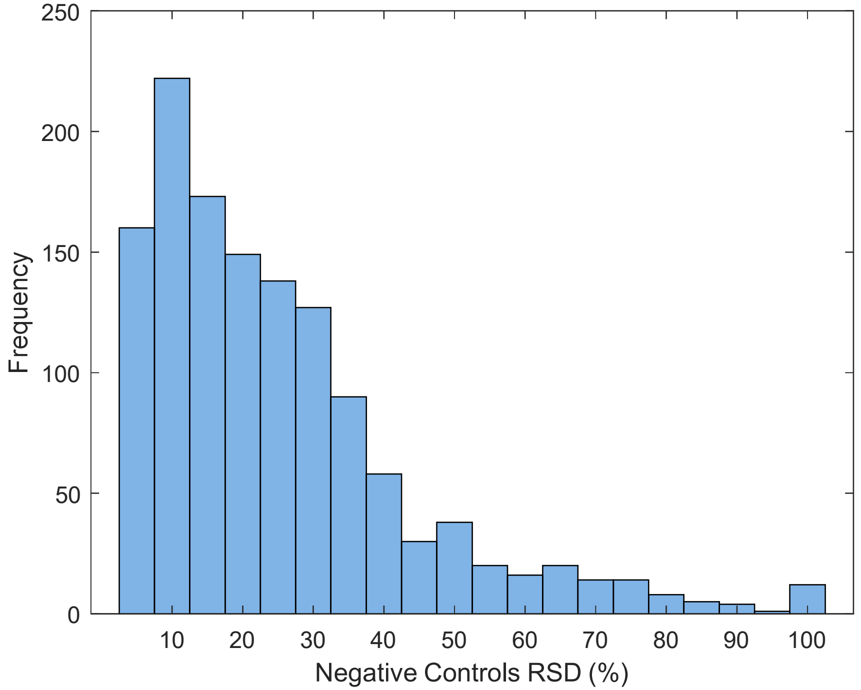

c)

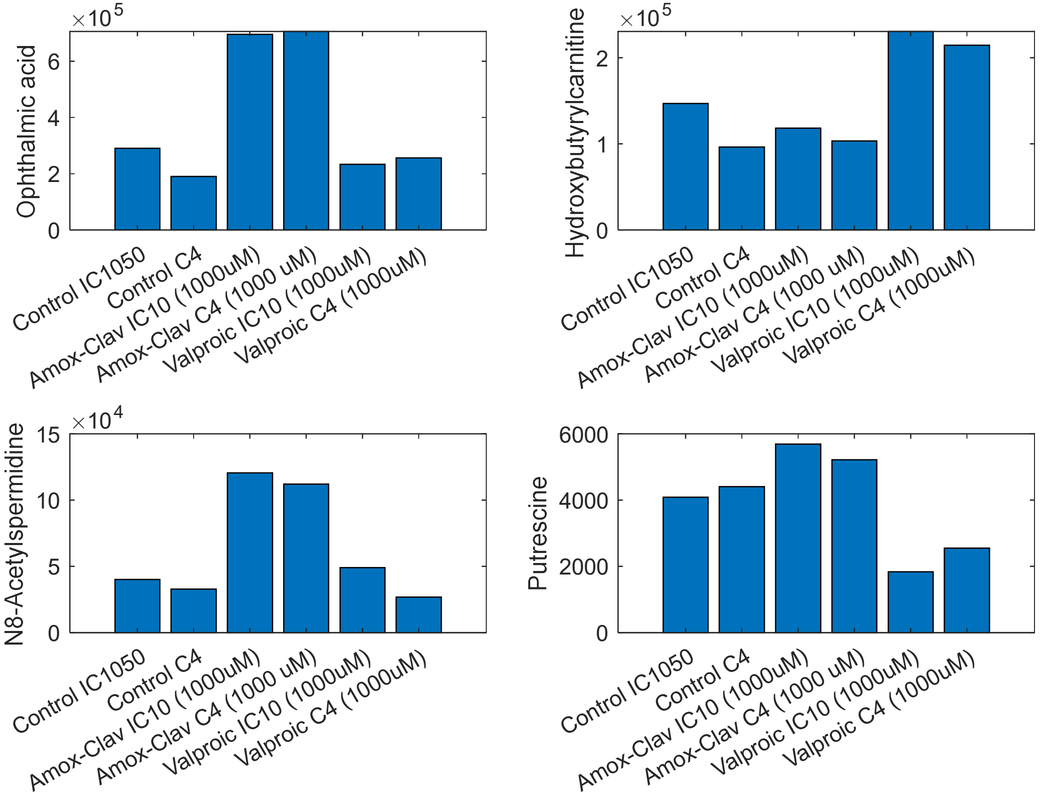

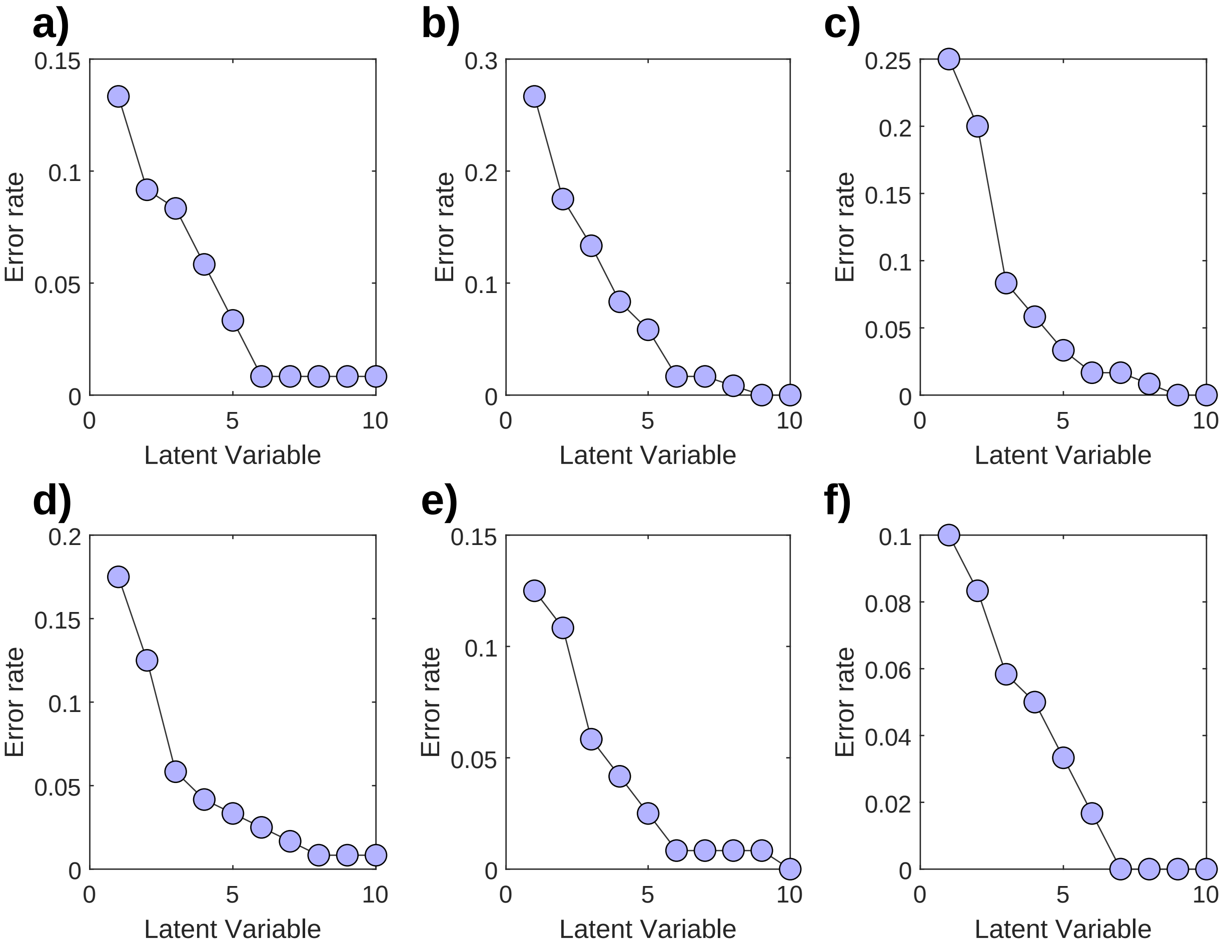
a)
b)
c)
d)
e)
f)
Error rate
Latent Variable

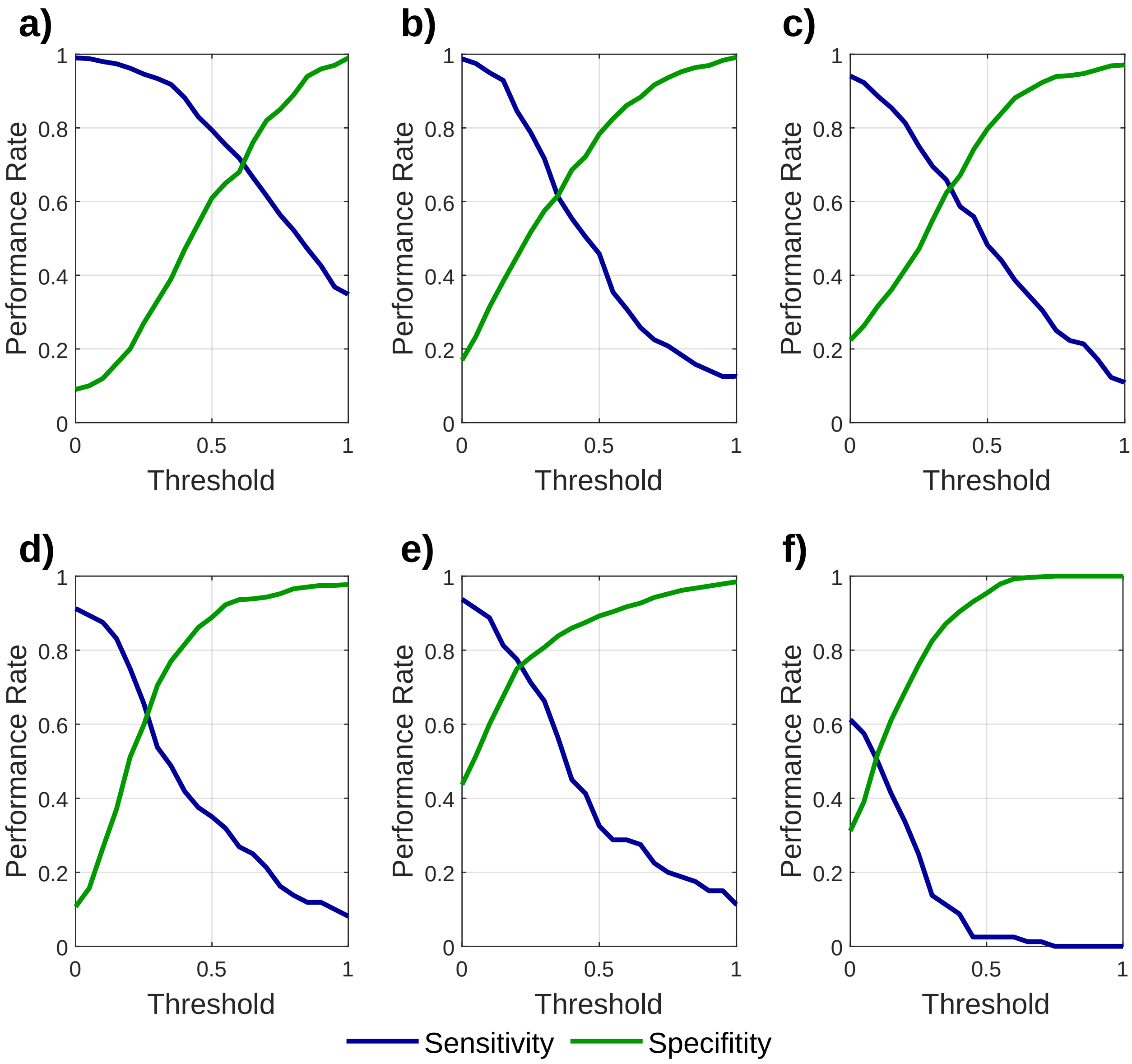

a)
b)
c)
d)
e)
f)
Performance Rate
Threshold
0
0.2
0.4
0.6
0.8
1
0.5
Sensitivity
Specifitity

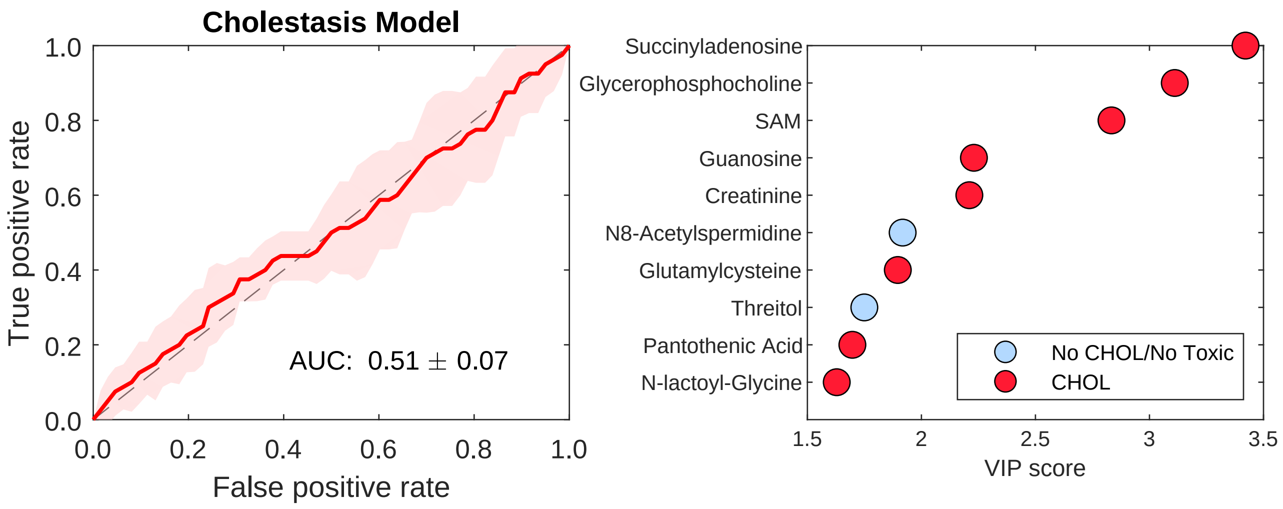

Cholestasis Model
True positive rate
False positive rate
0.0
0.2
0.4
0.6
0.8
1.0
AUC: 0.51 ± 0.07
Succinyladenosine
Glycerophosphocholine
SAM
Guanosine
Creatinine
N8-Acetylspermidine
Glutamylcysteine
Threitol
Pantothenic Acid
N-lactoyl-Glycine
No CHOL/No Toxic
CHOL
1.5
2
2.5
3
3.5
VIP score

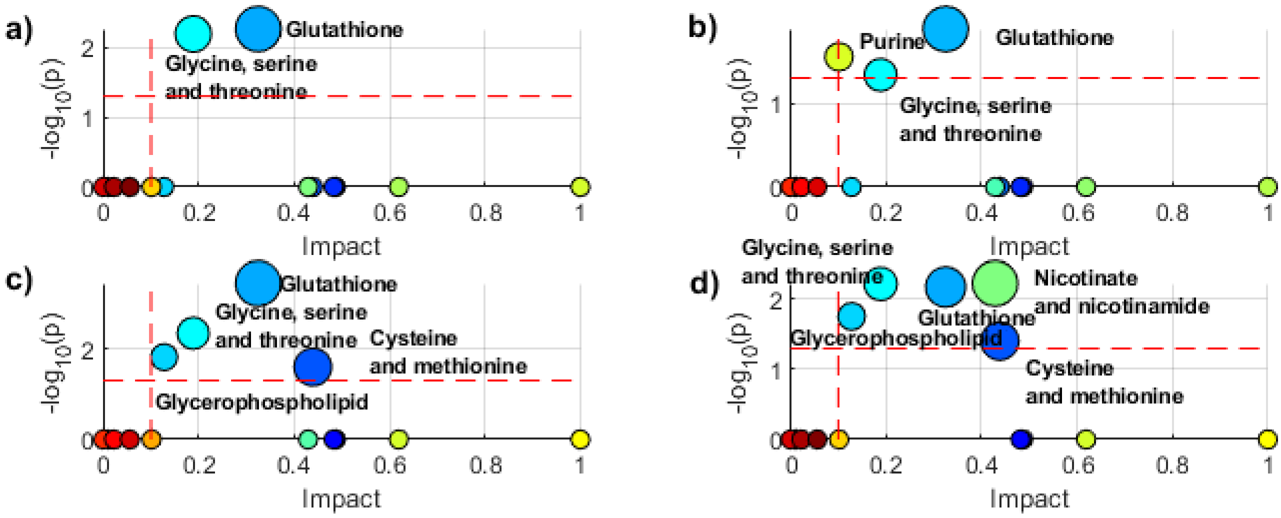

a)
Glutathione
Glycine, serine
and threonine
-log10(p)
Impact
b)
Purine
Glutathione
Glycine, serine
and threonine
-log10(p)
Impact
c)
Glutathione
Glycine, serine
and threonine
Cysteine
and methionine
Glycerophospholipid
-log10(p)
Impact
d)
Glycine, serine
and threonine
Nicotinate
and nicotinamide
Glutathione
Glycerophospholipid
Cysteine
and methionine
-log10(p)
Impact

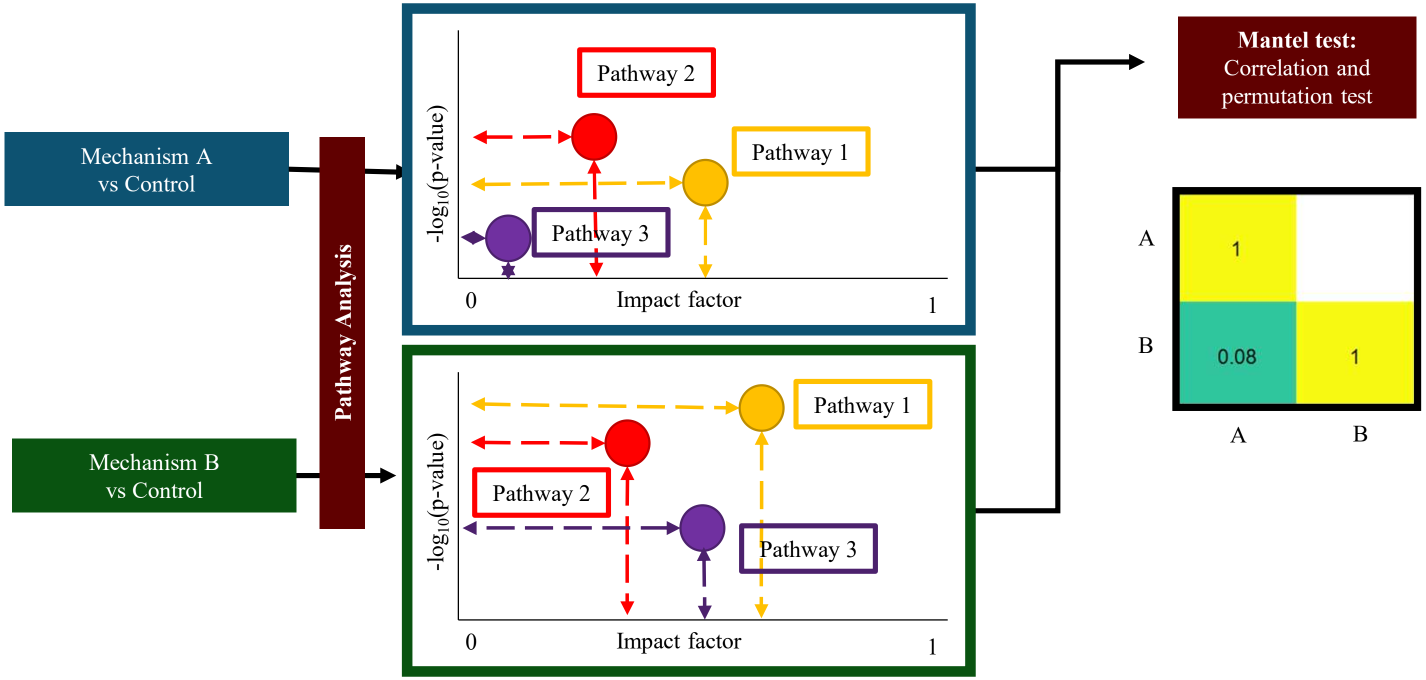

Mechanism A
vs Control
Mechanism B
vs Control
Pathway Analysis
Pathway 2
Pathway 1
Pathway 3
-log10(p-value)
0
Impact factor
1
Pathway 1
Pathway 2
Pathway 3
-log10(p-value)
0
Impact factor
1
Mantel test:
Correlation and
permutation test
A
B
1
0.08
1
A
B

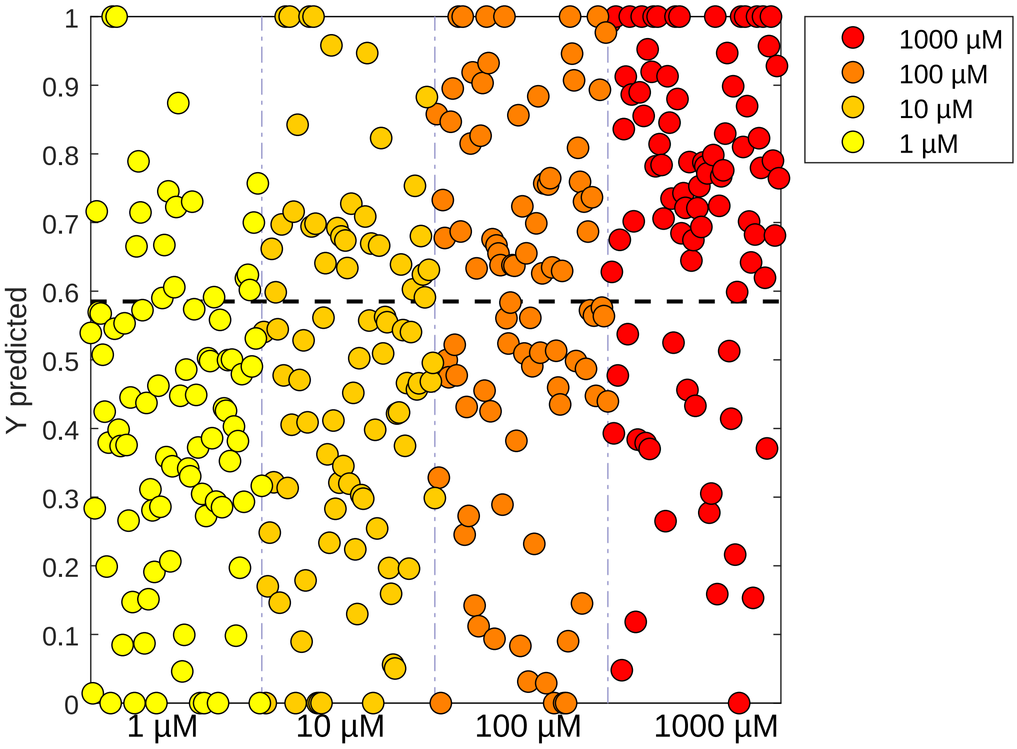

1
0.9
0.8
0.7
0.6
0.5
0.4
0.3
0.2
0.1
0
Y predicted
1 µM
10 µM
100 µM
1000 µM
1000 µM
100 µM
10 µM
1 µM

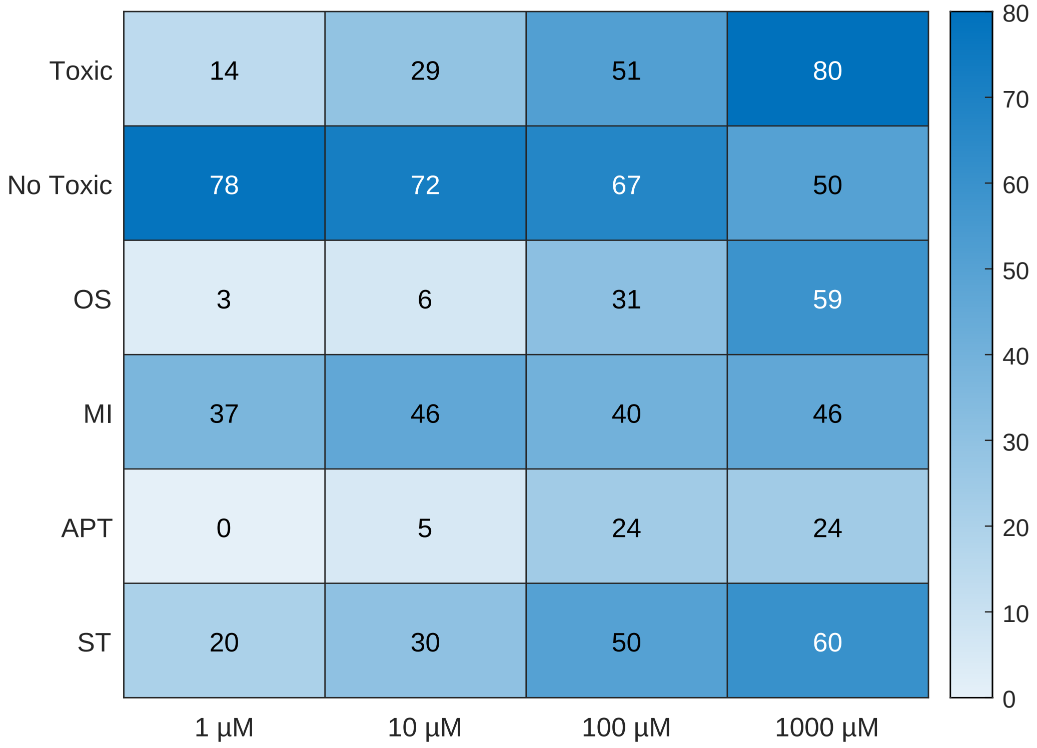
Toxic
14
29
51
80
No Toxic
78
72
67
50
OS
3
6
31
59
MI
37
46
40
46
APT
0
5
24
24
ST
20
30
50
60
1 µM
10 µM
100 µM
1000 µM
80
70
60
50
40
30
20
10
0

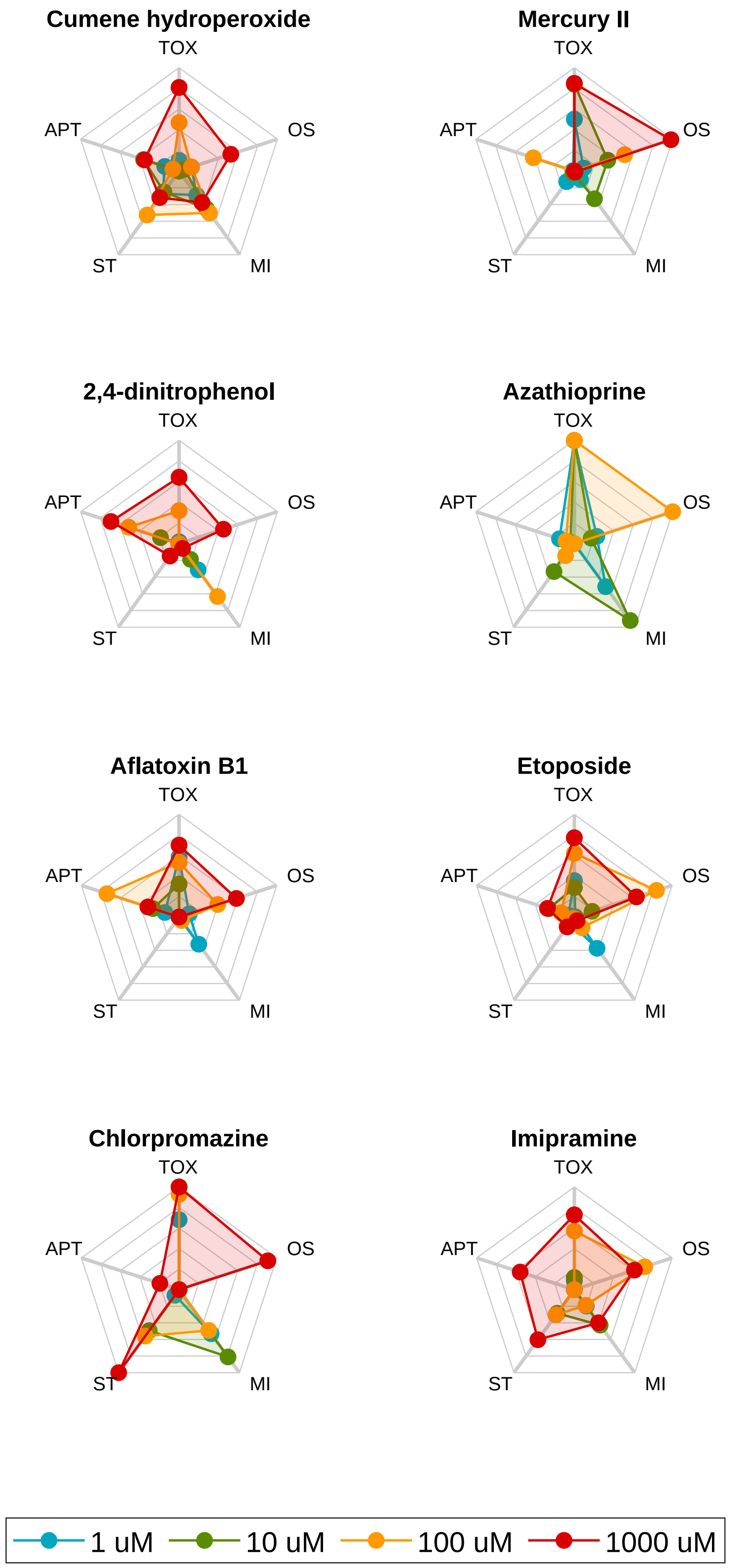

Cumene hydroperoxide
Mercury II
2,4-dinitrophenol
Azathioprine
Aflatoxin B1
Etoposide
Chlorpromazine
Imipramine
TOX
OS
MI
ST
APT
1 uM
10 uM
100 uM
1000 uM

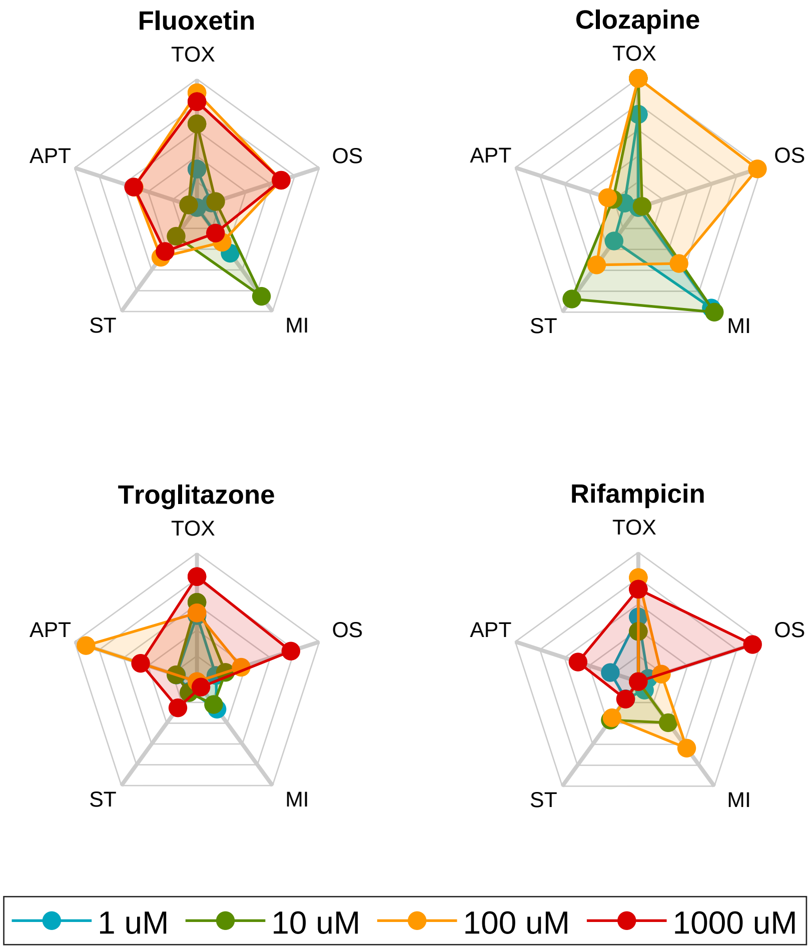

Fluoxetin
Clozapine
Troglitazone
Rifampicin
TOX
OS
MI
ST
APT
1 uM
10 uM
100 uM
1000 uM
