# Supplementary material for: Metabolomics-based strategy to assess drug hepatotoxicity and uncover the mechanisms of hepatotoxicity involved
Source: Arch Toxicol. 2023 Apr 6;97(6):1723–38. doi: 10.1007/s00204-023-03474-8 (PMC10182947; doi:10.1007/s00204-023-03474-8)
Supplement: Supplementary file 2 — Supplementary Table 1. List compounds of the test set. Compounds assayed are shown along with their CAS number. Four concentrations were investigated 1.10, 100 and the maximum indicated in the table (conditioned by the solubility in DMSO), and the stock solvent used. Supplementary Table 2. List of identified metabolites by untargeted metabolomics. All identified metabolites were shown including mass-to-charge ratio (m/z), retention time (RT), type of column (Synergy for Method 1, Acquity C18 for Method 2), HMDB code, metabolic pathway,VIP scores, and t-test p-value of each prediction model (TOX, OS, MI, APT, ST). VIP scores higher than 1 are shown as red or blue on whether its weight on the model is higher in the samples classified as mechanism positive or negative, respectively. p-value < 0.05 is statistically significant and coloured as green. Supplementary Table 3. Summary of the toxicity indexes for global and specific mechanism of toxicity for the test set of compounds analysed at each concentration, and the mechanisms described in the literature. [file 204_2023_3474_MOESM2_ESM.pdf]

STable 1

Compound	CAS	Max C (µM)	Stock Solvent	Compound	CAS	Max C (µM)	Stock Solvent
17-a ethynylestradiol	57-63-6	1000	DMSO	Gentamicin	1403-66-3	1000	water
2,4-dinitrophenol	51-28-5	1000	DMSO	Glucose	50-99-7	1000	water
3-acetamidophenol	621-42-1	1000	water	Glycochenodeoxycholate	16564-43-5	1000	DMSO
Acetaminophen	103-90-2	1000	water	Imipramine	50-49-7	1000	DMSO
Acetylsalicilic	50-78-2	1000	DMSO	Indomethacin	53-86-1	1000	DMSO
Aflatoxin B1	1162-65-8	1000	DMSO	Isoniazid	54-85-3	1000	water
Amikacin	37517-28-5	1000	water	Ketoconazole	65277-42-1	1000	DMSO
Amiodarone	1951-25-3	540	DMSO	Ketotifen	34580-14-8	1000	DMSO
Amitriptyline	549-18-8	1000	water	Lactose	10039-26-6	1000	water
Amoxicillin/clavulanate	26787-78-0 / 61177-45-5	1000/427	water	Levofloxacin	100986-85-4	1000	water
Ascorbic Acid	50-81-7	1000	water	Lovastatin	75330-75-5	1000	DMSO
Atorvastatin	134523-00-5	250	DMSO	Malathion	121-75-5	1000	DMSO
Atropine	51-55-8	1000	water	Maprotiline	10262-69-8	1000	DMSO
Azathioprine	446-86-6	800	DMSO	Menadione	58-27-5	1000	DMSO
Betaine	107-43-7	1000	water	Mercaptopurine	50-44-2	1000	DMSO
Bosentan	147536-97-8	1000	water	Mercury II	7487-94-7	1000	DMSO
Bupropion	34841-39-9	1000	water	Methotrexate	59-05-2	1000	DMSO
Buspirone	36505-84-7	1000	DMSO	N-acetylcysteine	616-91-1	1000	water
Caffeine	58-08-2	1000	water	NNK	64091-91-4	1000	DMSO
Captopril	62571-86-2	1000	water	Orphenadrine	83-98-7	1000	DMSO
Carbamazepine	298-46-4	1000	DMSO	Paraquat	4685-14-7	1000	water
Chloramphenicol	50-53-3	1000	DMSO	Pentachlorophenol	87-86-5	1000	water
Chloroquine	54-05-7	1000	water	Phenobarbital	50-06-6	1000	DMSO
Chlorpromazine	50-53-3	1000	DMSO	Phenytoin	57-41-0	1000	DMSO
Citrate	6132-04-3	1000	water	Piperonylbutoxide	51-03-6	1000	DMSO
Clozapine	5786-21-0	1000	DMSO	Propanolol	525-66-6	1000	DMSO
Colchicine	64-86-8	1000	water	Riboflavin	83-88-5	1000	water
Cumene hydroperoxide	80-15-9	1000	water	Rifampicin	13292-46-1	1000	water
Cycloheximide	66-81-9	1000	water	Simvastatin	79902-63-9	1000	DMSO
Cyclophosphamide	50-18-0	1000	water	Sorbitol	50-70-4	1000	water
Cyclosporine A	59865-13-3	300	DMSO	Stavudine	3056-17-5	1000	water
Dantrolene	7261-97-4	1000	DMSO	Tamoxifen	10540-29-1	510	DMSO
Dexamethasone	50-02-2	1000	DMSO	Taurolithocholate	6042-32-6	1000	DMSO
Diclofenac	15307-86-5	1000	water	Tetracycline	64-75-5	1000	water
Digoxin	20830-75-5	1000	DMSO	Thiabendazole	148-79-8	1000	DMSO
Dimethylsulfoxide	67-68-5	1000	-	Thiamine	67-03-8	1000	water
Erythromycin	114-07-8	1000	DMSO	Thioacetamide	62-55-5	1000	DMSO
Etoposide	33419-42-0	1000	DMSO	Ticlopidine	55142-85-3	1000	DMSO
Fenofibrate	49562-28-9	1000	DMSO	Tilorone	27591-69-1	1000	DMSO
Fialuridine	69123-98-4	500	DMSO	Troglitazone	97322-87-7	500	DMSO
Fluoxetine	54910-89-3	1000	water	Valproic	1069-66-5	1000	DMSO
Flutamide	13311-84-7	1000	DMSO	Verapamil	52-53-9	1000	water
Galactosamine	1772-03-8	1000	DMSO	Warfarin	81-81-2	1000	DMSO

STable 2

Metabolite	mz-RT	Column	HMDB	Metabolic Pathway	VIP score					p-value				
					TOX	OS	MI	APT	ST	TOX	OS	MI	APT	ST
Putrescine	89.1074-0.8	Synergy	HMDB0001414	Glutathione/Arginine and proline	1,41	0,33	1,68	2,42	1,15	0,68	0,96	0,83	0,01	0,67
Sarcosine	90.0548-1	Synergy	HMDB0000271	Glycine, serine and threonine	0,76	1,00	0,79	0,85	0,99	0,13	0,06	0,82	0,96	0,36
Choline	104.107-0.9	Synergy	HMDB0000097	Glycine, serine and threonine/Glycerophospholipid	1,86	2,47	1,73	2,66	2,03	0,04	0,04	0,98	0,02	0,21
Creatinine	114.0662-1	Synergy	HMDB0000562		0,98	1,55	1,86	0,50	1,56	0,03	0,33	0,08	0,60	0,02
Proline	116.0707-1.3	Synergy	HMDB0000162	Aminoacyl-tRNA /Arginine and proline	1,57	0,91	0,72	0,87	0,86	0,25	0,09	0,75	0,95	0,04
Valine	118.0864-1	Synergy	HMDB0000883	Aminoacyl-tRNA /Valine, leucine and isoleucine	0,66	1,16	0,93	0,42	0,45	0,14	<0.01	0,05	0,85	0,97
L-Threonine	120.0657-0.9	Synergy	HMDB0000167	Aminoacyl-tRNA /Valine, leucine and isoleucine	0,17	0,47	0,43	0,51	0,32	0,95	0,25	0,20	0,35	0,29
Cysteine	122.0269-1	Synergy	HMDB0000574	Aminoacyl-tRNA /Glutathione/Glycine, serine and threonine	2,63	1,43	1,61	0,72	1,88	<0.01	<0.01	0,88	0,19	0,04
Threitol	123.0554-1.8	Synergy	HMDB0004136		1,07	1,09	1,22	1,33	0,73	0,05	0,57	0,26	0,61	0,10
Niacin	124.0391-1.7	Synergy	HMDB0001406		1,01	1,19	1,71	1,26	1,23	0,72	0,25	0,10	0,35	0,24
Pyroglutamic	130.05-1.3	Synergy	HMDB0000267		2,08	1,58	1,20	1,19	1,22	<0.01	<0.01	0,40	0,05	0,14
Creatine	132.0768-1.2	Synergy	HMDB0000064	Arginine and proline/Glycine, serine and threonine	0,49	0,34	0,48	0,46	0,34	0,01	0,15	0,16	0,66	0,26
3-Methylindole	132.0808-4.9	Synergy	HMDB0000466		0,98	0,72	0,95	1,40	1,75	0,50	0,81	0,44	0,76	0,25
Leucine	132.102-2.5	Synergy	HMDB0000687	Aminoacyl-tRNA /Valine, leucine and isoleucine	0,94	0,54	0,90	0,66	0,52	0,18	0,91	0,12	0,68	0,77
L-Malic acid	135.0271-1.5	Synergy	HMDB0000156		0,11	0,06	0,12	0,03	0,18	0,64	0,29	0,60	0,77	0,71
Homocysteine	136.0484-1	Synergy	HMDB0000742	Cysteine and methionine	1,05	0,82	0,49	1,27	0,95	0,13	0,30	0,55	0,77	0,34
Adenine	136.0617-1.5	Synergy	HMDB0000034	Purine	0,33	0,25	0,22	0,20	0,18	0,27	0,41	0,82	0,51	0,71
Phenacylamine	136.0756-2.4	Synergy	HMDB0032628		0,58	0,73	0,68	0,62	0,53	0,59	0,16	0,21	0,74	0,35
2-Keto-glutaramic acid	146.0602-4.9	Synergy	HMDB0001552		0,63	0,84	1,32	0,96	0,94	0,39	0,48	0,06	0,90	0,63
Spermidine	146.1654-0.7	Synergy	HMDB0001257	Glutathione/Arginine and proline	0,20	0,23	0,28	0,17	0,23	0,15	0,31	0,34	0,84	0,24
Glutamine	147.0766-0.9	Synergy	HMDB0000641	Aminoacyl-tRNA /Purine	0,75	1,67	1,45	1,06	0,80	0,25	0,63	0,34	0,15	0,81
L-Glutamic acid	148.0608-0.9	Synergy	HMDB0000148	Glutathione/Arginine and proline	0,65	0,88	0,40	0,84	0,59	0,05	0,01	0,66	0,16	0,24
Methionine	150.0586-1.5	Synergy	HMDB0000696	Aminoacyl-tRNA	0,49	0,52	0,64	0,83	0,47	0,56	0,59	0,22	0,58	0,71
7-Methylhypoxanthine	151.0613-1.5	Synergy	HMDB0003162		0,23	0,36	0,53	0,42	0,40	0,62	0,46	0,16	0,96	0,98
Guanine	152.0549-1.5	Synergy	HMDB0000132	Purine	0,66	0,95	1,30	0,55	0,54	0,81	0,81	0,51	0,67	0,91
FAPy-adenine	154.0587-1	Synergy	HMDB0004816		0,79	0,78	0,41	1,37	0,71	0,08	0,15	0,51	0,63	0,22
N-Methylglutamic acid	162.0763-1.3	Synergy	HMDB0062660		1,75	1,58	0,91	1,05	1,07	<0.01	<0.01	0,50	0,05	0,12
Carnitine	162.1126-1	Synergy	HMDB0000062		1,89	2,07	0,65	0,94	1,24	<0.01	<0.01	0,85	0,35	0,04
2-Hydroxycinnamic acid	165.0544-2.4	Synergy	HMDB0002641		0,33	0,83	0,83	0,47	0,70	0,76	0,17	0,20	0,94	0,34
Arginine	175.1189-0.9	Synergy	HMDB0000517	Aminoacyl-tRNA /Arginine and proline	0,32	1,01	0,96	0,22	0,64	0,26	0,25	0,01	0,64	0,28
Citrulline	176.1029-1	Synergy	HMDB0000904	Arginine	0,51	1,05	0,96	0,79	0,59	0,45	<0.01	0,03	0,77	0,24
Gluconolactone	179.0484-1.6	Synergy	HMDB0000150		0,77	1,07	0,73	0,85	0,56	0,02	<0.01	0,28	0,43	0,07
Tyrosine	182.081-2.4	Synergy	HMDB0000158	Aminoacyl-tRNA /Phenylalanine, tyrosine and tryptophan	0,51	0,72	0,73	0,51	0,50	0,68	0,16	0,18	0,86	0,46
Phosphocholine	184.0736-0.9	Synergy	HMDB0001565	Glycerophospholipid	1,24	1,17	0,76	0,87	0,98	0,03	0,04	0,60	0,22	0,03
Phosphoserine	186.0164-0.9	Synergy	HMDB0000272	Cysteine and methionine/Glycine, serine and threonine	0,80	0,95	0,26	0,67	0,56	0,01	<0.01	0,98	0,13	0,06
Indoleacrylic acid	188.0708-4.9	Synergy	HMDB0000734		0,60	1,13	1,52	1,01	0,70	0,38	0,38	0,04	1,00	0,81
N8-Acetylspermidine	188.1758-0.9	Synergy	HMDB0002189		1,42	1,19	2,35	2,39	1,92	0,09	0,66	0,02	0,01	0,66
N-Acetylglutamic	190.0707-1.2	Synergy	HMDB0001138	Arginine	0,84	1,64	1,71	1,44	1,77	0,68	0,06	0,10	0,16	0,11
Spermine	203.2231-0.7	Synergy	HMDB0001256	Glutathione/Arginine and proline	0,32	0,30	0,20	0,26	0,32	0,04	0,05	0,69	0,19	0,13
Acetylcarnitine	204.1232-1.5	Synergy	HMDB0000201		0,71	0,39	0,18	0,63	0,32	0,04	0,15	0,89	0,36	0,20
Glutamyl-glycine	205.0815-1	Synergy	HMDB0028819		1,63	0,57	0,66	0,60	0,88	0,51	0,53	0,79	0,56	0,20
L-Tryptophan	205.0973-4.9	Synergy	HMDB0000929	Aminoacyl-tRNA	0,63	1,17	1,53	0,91	0,66	0,36	0,36	0,04	0,91	0,82
Pantothenic Acid	220.1183-4.5	Synergy	HMDB0000210		0,94	1,04	1,58	0,48	2,17	0,39	0,76	0,21	0,66	<0.01
Porphobilinogen	227.1025-4.5	Synergy	HMDB0000245		1,16	1,33	1,26	1,44	1,15	0,79	0,21	0,32	0,41	0,30
Cytidine	244.0926-1.4	Synergy	HMDB0000089		0,22	0,31	0,19	0,18	0,64	0,50	0,69	0,59	0,76	0,05
Glutamyl-threonine	249.108-1.2	Synergy	HMDB0028829		1,35	1,81	1,32	2,13	1,55	0,30	0,08	0,42	0,05	0,28
gamma-Glutamylcysteine	251.0694-1.6	Synergy	HMDB0001049	Glutathione	0,71	0,67	0,34	0,41	1,26	0,25	0,04	0,25	0,84	0,07
Glycerophosphocholine	258.1101-0.9	Synergy	HMDB0000086	Glycerophospholipid	1,84	2,26	2,01	1,85	1,39	0,59	0,05	0,19	0,38	0,80
Lysyl-Isoleucine	260.1968-1.3	Synergy	HMDB0028954		0,68	0,88	0,91	0,15	0,30	0,43	0,28	0,91	0,70	0,21
gamma-Glutamylleucine	261.1444-4.6	Synergy	HMDB0011171		1,45	1,03	0,91	1,56	1,13	0,57	0,74	0,30	0,41	0,24
L-phenylalanyl-L-proline	263.1463-1	Synergy	HMDB11177		1,07	0,51	0,90	0,61	0,58	0,02	0,26	0,13	0,80	0,20
Norophthalmic acid	276.1188-1.1	Synergy	HMDB0005766		0,51	0,95	0,73	0,44	0,62	0,27	0,52	0,65	0,58	0,11
Glutamyl-glutamic	277.1029-1.3	Synergy	HMDB0001138		0,40	0,69	0,17	0,32	0,45	0,28	0,09	0,47	0,26	0,28
Guanosine	284.0986-4	Synergy	HMDB0000133	Purine	3,01	1,23	1,15	1,97	1,10	0,06	0,67	0,50	0,42	0,41
N1,N12-Diacetylspermine	287.2439-1.1	Synergy	HMDB0002172		1,05	1,38	2,07	1,67	3,47	0,51	0,56	0,20	0,74	0,02
MTA	298.097-4.8	Synergy	HMDB0001173	Cysteine and methionine	0,01	0,01	0,01	0,01	0,02	0,89	0,88	0,95	0,78	0,24
Glutathione	308.0931-1.6	Synergy	HMDB0062697	Glutathione	0,69	1,17	1,20	0,73	0,80	0,07	<0.01	0,16	0,77	0,13
L-L-Homoglutathione	322.1062-2.5	Synergy	HMDB0062697		0,32	0,39	0,71	0,73	0,63	0,87	0,01	0,01	0,05	0,41
CMP	324.0587-1.1	Synergy	HMDB0029395		0,29	0,63	0,31	0,33	0,26	0,87	0,55	0,43	0,45	0,12
cAMP	330.0727-1.6	Synergy	HMDB0000095		0,77	1,76	1,06	0,26	0,82	0,05	<0.01	0,09	0,76	0,19
gamma-Glutamylcysteinylserine	338.1013-1.5	Synergy	HMDB0039733		0,10	0,13	0,12	0,07	0,06	0,04	0,02	0,50	0,67	0,07
Formylmethyl-glutathione	350.1013-4.1	Synergy	HMDB0060507		0,34	0,28	0,29	0,54	0,19	0,03	0,47	0,24	0,28	0,11
Succinyladenosine	384.1146-4.5	Synergy	HMDB0000912		1,31	2,04	2,02	1,70	1,62	0,52	0,24	0,62	0,63	0,56
Deoxycholic acid	393.2856-9.5	Synergy	HMDB0000626		1,33	0,56	0,42	0,92	0,48	0,29	0,76	0,75	0,19	0,95
SAM	399.1434-0.9	Synergy	HMDB0001185	Arginine and proline/Cysteine and methionine	1,45	0,63	0,84	2,38	1,38	0,91	0,41	0,46	0,02	0,77
Cysteineglutathione disulfide	427.0946-1	Synergy	HMDB0000656		0,50	0,75	0,86	0,33	0,59	0,87	0,18	0,42	0,46	0,21
CDP-Ethanolamine	447.0669-1	Synergy	HMDB0001564	Glycerophospholipid	0,60	0,43	0,66	0,21	0,20	0,88	0,22	0,59	0,81	0,14
Acetamidopropanal	116.0695-0.9	Acquity	HMDB0012880		0,89	0,54	0,91	0,55	0,87	0,02	0,54	0,13	0,56	0,02
Proline	116.0704-0.7	Acquity	HMDB0003411		0,53	0,47	0,44	0,44	0,66	0,08	0,45	0,43	0,47	0,02
Indole	118.0644-2.7	Acquity	HMDB0000738		0,22	0,06	0,11	0,23	0,09	0,31	0,90	0,49	0,15	0,49
Betaine	118.0853-0.7	Acquity	HMDB0000043	Glycine, serine and threonine	0,56	1,89	2,24	0,64	1,25	0,60	0,02	0,02	0,74	0,21
Methylmalonic acid	119.0487-1	Acquity	HMDB0000202		0,27	0,22	0,19	0,22	0,35	0,58	0,87	0,65	0,33	0,02
Niacinamide	123.0543-0.9	Acquity	HMDB0001406		0,32	0,15	0,16	0,14	0,29	0,18	0,61	0,31	0,84	0,03
Nicotinic acid	124.0465-1	Acquity	HMDB0001488		0,45	0,37	0,26	0,38	0,51	0,70	0,92	0,74	0,29	0,02
4-Oxoproline	130.0489-0.6	Acquity	HMDB0304793	Glutathione	1,15	0,73	0,73	1,01	0,88	0,50	0,93	0,68	0,86	0,04
N-Acryloylglycine	130.0492-0.8	Acquity	HMDB0001843		2,39	0,81	1,75	0,41	0,71	<0.01	0,95	0,06	0,63	0,03
3-Methylindole	132.0799-2.7	Acquity	HMDB0000466		0,03	0,01	0,02	0,02	0,02	0,49	0,91	0,55	0,33	0,40
Isoleucine	132.1016-1.3	Acquity	HMDB0000172	Aminoacyl-tRNA /Valine, leucine and isoleucine	0,61	0,22	0,30	0,39	0,41	0,67	0,67	0,54	0,30	0,15
Ornithine	133.1044-1.2	Acquity	HMDB0000214	Glutathione/Arginine and proline	0,85	0,37	0,32	0,58	0,81	0,90	0,63	0,65	0,27	0,06
2-Phenylacetamide	136.0605-1	Acquity	HMDB0010715		0,05	0,03	0,02	0,03	0,15	0,23	0,41	0,92	0,68	0,20
Hypoxanthine	137.059-1	Acquity	HMDB0000157		0,59	0,52	0,76	0,30	0,41	0,31	0,36	0,04	0,92	0,88
4-Guanidinobutanoate	146.0917-0.8	Acquity	HMDB0003464	Arginine and proline	1,52	0,89	0,47	0,74	1,02	0,02	0,31	0,46	0,73	0,04
N-lactoyl-Glycine	148.0594-1	Acquity	HMDB0062183		1,49	1,14	2,38	1,06	1,43	0,22	0,59	0,04	0,65	0,15
L-Histidine	156.0759-0.7	Acquity	HMDB0000177	Aminoacyl-tRNA	0,49	0,24	0,29	0,39	0,49	0,41	0,94	0,52	0,25	0,14
8-Amino Caprylic acid	160.1324-0.8	Acquity	NA		1,23	0,87	0,77	0,67	1,28	0,02	0,45	0,24	0,91	0,01
Phenylpyruvic acid	165.0547-1	Acquity	HMDB0000205	Phenylalanine, tyrosine and tryptophan	0,44	0,52	0,49	0,41	0,75	0,41	0,63	0,71	0,48	0,03
L-Phenylalanine	166.0872-2	Acquity	HMDB0000159	Aminoacyl-tRNA	0,24	0,24	0,23	0,25	0,33	0,64	0,67	0,87	0,49	0,05
5-Hydroxyindoleacetaldehyde	176.0698-6.1	Acquity	HMDB0004073		0,06	0,13	0,14	0,23	0,30	0,67	0,90	0,78	0,98	0,97
Tyrosine	182.0824-1	Acquity	HMDB0006050		0,37	0,48	0,38	0,48	0,67	0,35	0,48	0,80	0,68	0,03
Indoleacrylic acid	188.0702-2.7	Acquity	HMDB0000734		0,08	0,04	0,05	0,06	0,05	0,73	0,80	0,72	0,28	0,22
N1-Acetylspermidine	188.1737-0.6	Acquity	HMDB0001276		1,13	0,84	0,64	1,75	1,36	0,07	0,36	0,44	<0.01	0,77
N-Acetyl-L-Histidine	198.0862-0.7	Acquity	HMDB0032055		0,59	0,33	0,35	1,25	2,43	0,42	0,93	0,62	0,35	<0.01
Tryptophan	205.0963-2.7	Acquity	HMDB0000929	Aminoacyl-tRNA	0,15	0,07	0,09	0,13	0,09	0,63	0,81	0,62	0,24	0,26
Xanthurenic acid	206.0535-0.6	Acquity	HMDB0000881		1,05	0,94	0,75	1,01	0,94	0,04	0,52	0,20	0,35	0,03
Propionylcarnitine	218.138-1.5	Acquity	HMDB0000824		1,15	2,43	1,01	2,35	1,26	0,05	0,71	0,07	0,33	0,01
Cysteinyl-Proline	219.0793-2.3	Acquity	HMDB0028783		1,18	0,52	0,63	0,20	0,25	0,30	0,88	0,53	0,91	0,47
N1,N8-Diacetylspermidine	230.1855-1	Acquity	HMDB0041947		0,92	0,76	0,72	1,56	1,18	0,13	0,07	0,68	<0.01	0,53
Butyryl-carnitine	232.1536-2.5	Acquity	HMDB0002013		1,38	0,38	0,69	0,54	0,80	<0.01	0,32	0,05	0,90	<0.01
Histidylproline	235.1184-1	Acquity	HMDB0028893		0,92	2,07	1,37	0,31	0,71	0,36	0,53	0,12	0,84	0,10
N-acetylspermine	245.232-0.6	Acquity	HMDB0001186		0,34	0,21	0,19	0,47	0,39	0,14	0,31	0,62	0,01	0,35
Glutamyl-valine	247.1282-2	Acquity	HMDB0028832		0,18	0,18	0,11	0,32	0,21	0,34	0,14	0,33	0,02	0,18
Hydroxybutyrylcarnitine	248.1483-1	Acquity	HMDB0013127		1,23	1,68	1,84	0,60	2,43	0,02	0,04	0,74	0,46	0,72
Glutamylcysteine	249.0529-1.2	Acquity	HMDB0028816		1,96	0,99	1,43	1,92	1,42	0,97	0,51	0,75	0,36	0,20
Adenosine	268.1033-1	Acquity	HMDB0000050	Purine	0,05	0,09	0,08	0,07	0,06	0,33	0,13	0,29	0,25	0,49
Ophthalmic acid	290.1338-0.9	Acquity	HMDB0005765	Cysteine and methionine	1,67	1,88	1,68	2,40	1,39	0,55	0,23	0,32	0,03	0,11
Cyclic AMP	330.0724-0.9	Acquity	HMDB0000058		0,82	0,64	1,30	0,42	0,74	0,05	0,08	0,68	0,47	0,05
Cyclic GMP	346.0455-0.9	Acquity	HMDB0001314	Purine	0,00	0,00	0,00	0,00	0,00	<0.01	0,19	0,20	0,37	<0.01
dGMP	348.0693-0.9	Acquity	HMDB0001044		0,22	0,38	0,34	0,45	0,29	0,80	0,25	0,28	0,29	0,53
AMP	348.0694-0.7	Acquity	HMDB0000045	Purine	1,42	2,56	2,19	2,42	1,10	0,36	0,08	0,31	0,12	0,38
S-Lactoylglutathione	380.1112-1	Acquity	HMDB0001066		0,12	0,14	0,09	0,13	0,12	0,04	0,61	0,16	0,28	0,03
SAH	385.128-0.9	Acquity	HMDB0000939		0,62	0,49	0,27	0,94	0,71	0,08	0,15	0,90	0,09	0,38
LysoPC(14:0)	468.3078-7.5	Acquity	HMDB0010379		0,06	0,09	0,02	0,05	0,03	0,06	0,20	0,65	0,33	0,13
LysoPC(16:1)	494.323-7.5	Acquity	HMDB0010383		0,33	0,39	0,24	0,41	0,26	0,90	0,43	0,31	0,73	0,90
LysoPC(16:0)	496.3391-8	Acquity	HMDB0010382		0,69	0,41	0,48	0,71	1,91	0,22	0,91	0,24	0,38	<0.01
LysoPC(18:1)	522.3546-8	Acquity	HMDB0002815		0,12	0,05	0,10	0,12	0,39	0,30	0,80	0,20	0,94	<0.01
NAD	664.1162-0.7	Acquity	HMDB0000902		1,21	0,90	1,17	0,68	1,07	0,13	0,88	0,32	0,62	0,01

STable3

order	Compound	Concentration (µM)	TOX Index	OS Index	MI Index	APT Index	ST Index	Bibliography Mechanism
1	17-a ethynylestradiol	1	0,54	0,25	0,39	0,22	0,18	MI,CHOL
2	17-a ethynylestradiol	10	0,54	0,32	0,24	0,13	0,11	MI,CHOL
3	17-a ethynylestradiol	100	0,89	0,06	1,00	0,00	0,43	MI,CHOL
4	17-a ethynylestradiol	1000	1,00	0,11	1,00	0,22	0,43	MI,CHOL
5	2,4-dinitrophenol	1	0,02	0,00	0,31	0,00	0,00	MI
6	2,4-dinitrophenol	10	0,00	0,00	0,19	0,19	0,00	MI
7	2,4-dinitrophenol	100	0,32	0,00	0,63	0,51	0,01	MI
8	2,4-dinitrophenol	1000	0,64	0,45	0,06	0,69	0,15	MI
9	3-acetamidophenol	1	0,28	0,00	0,41	0,00	0,00	NT
10	3-acetamidophenol	10	0,18	0,00	0,43	0,34	0,13	NT
11	3-acetamidophenol	100	0,00	0,00	0,00	0,00	0,00	NT
12	3-acetamidophenol	1000	0,39	0,23	0,06	0,03	0,22	NT
13	Acetaminophen	1	0,71	0,19	0,57	0,07	0,10	OS, AP
14	Acetaminophen	10	0,22	0,00	0,09	0,17	0,00	OS, AP
15	Acetaminophen	100	0,73	0,00	1,00	0,00	0,16	OS, AP
16	Acetaminophen	1000	1,00	0,00	1,00	0,00	0,56	OS, AP
17	Acetylsalicilic	1	0,58	0,14	0,27	0,00	0,00	OS, MI, ST
18	Acetylsalicilic	10	0,67	0,17	0,27	0,18	0,00	OS, MI, ST
19	Acetylsalicilic	100	0,69	0,18	0,13	0,28	0,00	OS, MI, ST
20	Acetylsalicilic	1000	0,51	0,71	0,00	0,00	0,20	OS, MI, ST
21	Aflatoxin B1	1	0,59	0,10	0,33	0,15	0,00	OS, AP
22	Aflatoxin B1	10	0,32	0,00	0,00	0,27	0,00	OS, AP
23	Aflatoxin B1	100	0,53	0,40	0,04	0,74	0,00	OS, AP
24	Aflatoxin B1	1000	0,70	0,59	0,00	0,32	0,00	OS, AP
25	Amikacin	1	0,51	0,00	0,56	0,00	0,17	
26	Amikacin	10	0,56	0,05	0,15	0,01	0,04	
27	Amikacin	100	0,45	0,03	0,00	0,08	0,00	
28	Amikacin	1000	0,03	0,00	0,00	0,04	0,05	
29	Amiodarone	1	0,38	0,14	0,38	0,00	0,00	MI, ST
30	Amiodarone	10	0,52	0,20	0,58	0,18	0,17	MI, ST
31	Amiodarone	100	0,86	0,28	0,73	0,36	0,70	MI, ST
32	Amiodarone	540	LP	LP	LP	LP	LP	MI, ST
33	Amitriptyline	1	0,21	0,01	0,28	0,05	0,08	OS, MI,CHOL
34	Amitriptyline	10	0,13	0,00	0,21	0,03	0,09	OS, MI,CHOL
35	Amitriptyline	100	LP	LP	LP	LP	LP	OS, MI,CHOL
36	Amitriptyline	1000	LP	LP	LP	LP	LP	OS, MI,CHOL
37	Amox/clav (4:1)	1/0.4	0,32	0,07	0,47	0,06	0,00	OS, CHOL
38	Amox/clav (4:1)	10/4	0,65	0,10	0,82	0,06	0,20	OS, CHOL
39	Amox/clav (4:1)	100/43	0,47	0,11	0,59	0,00	0,00	OS, CHOL
40	Amox/clav (4:1)	1000/427	0,87	0,18	0,95	0,35	0,15	OS, CHOL
41	Ascorbic Acid	1	0,00	0,05	0,39	0,28	0,20	NT
42	Ascorbic Acid	10	0,48	0,15	0,54	0,08	0,36	NT
43	Ascorbic Acid	100	0,49	0,21	0,44	0,06	0,38	NT
44	Ascorbic Acid	1000	0,61	0,31	0,65	0,11	0,40	NT
45	Atorvastatin	1	1,00	0,06	1,00	0,08	0,52	MI
46	Atorvastatin	10	1,00	0,15	1,00	0,18	0,66	MI
47	Atorvastatin	100	1,00	0,19	1,00	0,09	0,98	MI
48	Atorvastatin	250	1,00	0,43	1,00	0,00	0,85	MI
49	Atropine	1	0,54	0,19	0,46	0,27	0,25	
50	Atropine	10	0,31	0,18	0,32	0,35	0,20	
51	Atropine	100	0,71	0,22	0,54	0,00	0,27	
52	Atropine	1000	0,91	0,30	0,64	0,00	0,30	
53	Azathioprine	1	1,00	0,23	0,52	0,15	0,00	OS, MI, AP,CHOL
54	Azathioprine	10	1,00	0,17	0,92	0,04	0,33	OS, MI, AP,CHOL
55	Azathioprine	100	1,00	1,00	0,00	0,09	0,14	OS, MI, AP,CHOL
56	Azathioprine	800	LP	LP	LP	LP	LP	OS, MI, AP,CHOL
57	Betaine	1	0,39	0,17	0,47	0,32	0,23	NT
58	Betaine	10	0,43	0,06	0,39	0,15	0,24	NT
59	Betaine	100	0,25	0,00	0,47	0,16	0,20	NT
60	Betaine	1000	0,12	0,00	1,00	0,12	0,38	NT
61	Bosentan	1	0,37	0,00	0,31	0,00	0,00	MI, CHOL
62	Bosentan	10	0,71	0,04	0,39	0,11	0,09	MI, CHOL
63	Bosentan	100	0,44	0,67	0,06	0,00	0,20	MI, CHOL
64	Bosentan	1000	0,41	0,00	0,36	0,42	0,00	MI, CHOL
65	Bupropion	1	0,11	0,14	0,35	0,00	0,20	OS, AP
66	Bupropion	10	0,00	0,01	0,35	0,10	0,09	OS, AP
67	Bupropion	100	0,27	0,08	0,51	0,00	0,17	OS, AP
68	Bupropion	1000	0,90	0,27	0,79	0,01	0,35	OS, AP
69	Buspirone	1	0,54	0,00	0,00	0,14	0,26	
70	Buspirone	10	0,82	0,00	0,22	0,19	0,43	
71	Buspirone	100	0,83	0,23	0,17	0,00	0,17	
72	Buspirone	1000	1,00	0,04	0,55	0,24	0,40	
73	Caffeine	1	0,38	0,00	0,46	0,00	0,00	NT
74	Caffeine	10	0,48	0,00	0,41	0,00	0,00	NT
75	Caffeine	100	0,93	0,04	0,81	0,00	0,00	NT
76	Caffeine	1000	0,86	0,98	0,09	0,01	0,22	NT
77	Captopril	1	0,28	0,00	0,40	0,17	0,00	AP
78	Captopril	10	0,11	0,00	0,20	0,03	0,00	AP
79	Captopril	100	0,17	0,00	0,00	0,05	0,00	AP
80	Captopril	1000	0,40	0,00	0,25	0,12	0,00	AP
81	Carbamazepine	1	0,46	0,00	0,29	0,06	0,03	MI
82	Carbamazepine	10	0,53	0,03	0,00	0,08	0,08	MI
83	Carbamazepine	100	0,64	0,00	0,18	0,14	0,18	MI
84	Carbamazepine	1000	0,96	0,18	0,36	0,05	0,28	MI
85	Chloramphenicol	1	0,16	0,15	0,29	0,01	0,07	OS, MI
86	Chloramphenicol	10	0,21	0,18	0,65	0,00	0,29	OS, MI
87	Chloramphenicol	100	0,14	0,00	0,74	0,00	0,56	OS, MI
88	Chloramphenicol	1000	0,37	0,00	1,00	0,00	0,46	OS, MI
89	Chloroquine	1	0,00	0,11	0,07	0,00	0,09	OS, MI, AP

STable3

order	Compound	Concentration (µM)	TOX Index	OS Index	MI Index	APT Index	ST Index	Bibliography Mechanism
90	Chloroquine	10	0,40	0,20	0,35	0,20	0,24	OS, MI, AP
91	Chloroquine	100	0,86	0,38	0,55	0,30	0,48	OS, MI, AP
92	Chloroquine	1000	LP	LP	LP	LP	LP	OS, MI, AP
93	Chlorpromazine	1	0,68	0,00	0,53	0,00	0,07	OS, MI, CHOL
94	Chlorpromazine	10	1,00	0,00	0,81	0,00	0,49	OS, MI, CHOL
95	Chlorpromazine	100	0,93	0,00	0,49	0,00	0,56	OS, MI, CHOL
96	Chlorpromazine	1000	1,00	0,91	0,00	0,20	1,00	OS, MI, CHOL
97	Citrate	1	0,77	0,14	0,03	0,02	0,02	NT
98	Citrate	10	0,67	0,36	0,62	0,23	0,00	NT
99	Citrate	100	0,45	0,19	0,49	0,13	0,14	NT
100	Citrate	1000	0,77	0,30	0,58	0,11	0,18	NT
101	Clozapine	1	0,72	0,00	0,96	0,11	0,32	OS, MI
102	Clozapine	10	1,00	0,03	1,00	0,21	0,87	OS, MI
103	Clozapine	100	1,00	0,97	0,53	0,25	0,55	OS, MI
104	Clozapine	1000	LP	LP	LP	LP	LP	OS, MI
105	Colchicine	1	0,56	0,24	0,37	0,49	0,18	OS
106	Colchicine	10	0,70	0,23	0,53	0,52	0,17	OS
107	Colchicine	100	0,92	0,39	0,57	0,51	0,29	OS
108	Colchicine	1000	0,83	0,40	0,44	0,50	0,35	OS
109	Cumene hydroperoxide	1	0,10	0,13	0,29	0,15	0,27	OS
110	Cumene hydroperoxide	10	0,00	0,05	0,45	0,36	0,25	OS
111	Cumene hydroperoxide	100	0,47	0,13	0,50	0,06	0,53	OS
112	Cumene hydroperoxide	1000	0,81	0,53	0,38	0,35	0,32	OS
113	Cycloheximide	1	0,42	0,25	0,47	0,12	0,44	AP
114	Cycloheximide	10	0,00	0,24	0,00	0,01	0,10	AP
115	Cycloheximide	100	0,63	0,33	0,34	0,00	0,52	AP
116	Cycloheximide	1000	0,66	0,27	0,60	0,00	0,58	AP
117	Cyclophosphamide	1	0,15	0,00	0,35	0,09	0,06	
118	Cyclophosphamide	10	0,00	0,00	0,16	0,07	0,00	
119	Cyclophosphamide	100	0,12	0,00	0,22	0,11	0,07	
120	Cyclophosphamide	1000	0,25	0,00	0,38	0,08	0,08	
121	Cyclosporine A	1	0,30	0,03	0,00	0,15	0,04	MI
122	Cyclosporine A	10	0,56	0,03	0,00	0,00	0,21	MI
123	Cyclosporine A	100	0,69	0,09	0,00	0,00	0,31	MI
124	Cyclosporine A	300	0,89	0,30	0,00	0,08	0,27	MI
125	Dantrolene	1	0,33	0,00	0,42	0,09	0,00	OS
126	Dantrolene	10	0,67	0,57	0,20	0,11	0,25	OS
127	Dantrolene	100	0,68	0,00	0,26	0,22	0,21	OS
128	Dantrolene	1000	0,88	0,15	0,28	0,13	0,29	OS
129	Dexamethasone	1	0,22	0,00	0,52	0,10	0,06	NT
130	Dexamethasone	10	0,36	0,00	0,35	0,10	0,05	NT
131	Dexamethasone	100	0,65	0,00	0,50	0,00	0,40	NT
132	Dexamethasone	1000	0,75	0,25	0,27	0,00	0,24	NT
133	Diclofenac	1	0,00	0,00	0,38	0,10	0,00	MI, AP
134	Diclofenac	10	0,22	0,00	0,25	0,06	0,00	MI, AP
135	Diclofenac	100	0,26	0,06	0,33	0,20	0,04	MI, AP
136	Diclofenac	1000	0,50	0,00	0,91	0,00	1,00	MI, AP
137	Digoxin	1	0,42	0,81	0,13	0,00	1,00	MI
138	Digoxin	10	0,96	0,76	0,24	0,01	0,61	MI
139	Digoxin	100	1,00	0,65	0,54	0,00	1,00	MI
140	Digoxin	1000	1,00	0,75	0,28	0,00	0,94	MI
141	Dimethylsulfoxide	1	0,27	0,00	0,00	0,07	0,19	NT
142	Dimethylsulfoxide	10	0,39	0,20	0,11	0,38	0,20	NT
143	Dimethylsulfoxide	100	0,55	0,27	0,14	0,30	0,20	NT
144	Dimethylsulfoxide	1000	0,87	0,62	0,00	0,25	0,22	NT
145	Doxycycline	1	0,59	0,07	0,49	0,00	0,30	
146	Doxycycline	10	0,28	0,00	0,69	0,00	0,26	
147	Doxycycline	100	0,48	0,00	0,81	0,24	0,49	
148	Doxycycline	1000	1,00	0,85	0,00	0,24	0,55	
149	Erythromycin	1	0,67	0,78	0,00	0,01	0,15	OS, AP
150	Erythromycin	10	0,70	0,81	0,00	0,05	0,17	OS, AP
151	Erythromycin	100	0,61	0,04	0,19	0,13	0,27	OS, AP
152	Erythromycin	1000	0,72	0,35	0,05	0,07	0,16	OS, AP
153	Etoposide	1	0,36	0,01	0,38	0,06	0,07	AP
154	Etoposide	10	0,29	0,18	0,02	0,26	0,05	AP
155	Etoposide	100	0,62	0,84	0,13	0,14	0,02	AP
156	Etoposide	1000	0,77	0,64	0,04	0,28	0,12	AP
157	Fenofibrate	1	0,74	0,10	0,76	0,00	0,13	MI, AP
158	Fenofibrate	10	0,68	0,09	0,56	0,00	0,16	MI, AP
159	Fenofibrate	100	0,64	0,14	0,28	0,00	0,32	MI, AP
160	Fenofibrate	1000	0,73	0,35	0,23	0,00	0,37	MI, AP
161	Fialuridine	1	0,20	0,00	0,00	0,11	0,11	MI, ST
162	Fialuridine	10	0,34	0,00	0,00	0,11	0,03	MI, ST
163	Fialuridine	100	0,35	0,03	0,00	0,22	0,00	MI, ST
164	Fialuridine	500	0,42	0,31	0,00	0,17	0,00	MI, ST
165	Fluoxetine	1	0,30	0,12	0,44	0,07	0,00	OS
166	Fluoxetine	10	0,65	0,15	0,85	0,07	0,28	OS
167	Fluoxetine	100	0,89	0,69	0,33	0,51	0,48	OS
168	Fluoxetine	1000	0,82	0,69	0,25	0,52	0,42	OS
169	Flutamide	1	0,61	0,11	0,31	0,01	0,17	MI,CHOL
170	Flutamide	10	0,62	0,23	0,34	0,14	0,08	MI,CHOL
171	Flutamide	100	0,12	0,00	0,11	0,33	0,09	MI,CHOL
172	Flutamide	1000	0,64	0,00	0,88	0,30	0,61	MI,CHOL
173	Galactosamine	1	0,70	0,06	0,74	0,00	0,15	AP
174	Galactosamine	10	0,32	0,00	0,53	0,15	0,22	AP
175	Galactosamine	100	0,71	0,11	0,58	0,06	0,18	AP
176	Galactosamine	1000	0,66	0,27	0,35	0,22	0,24	AP
177	Gentamicin	1	0,86	0,18	0,79	0,02	0,05	NT
178	Gentamicin	10	0,71	0,11	0,56	0,00	0,02	NT

order	Compound	Concentration (µM)	TOX Index	OS Index	MI Index	APT Index	ST Index	Bibliography Mechanism
179	Gentamicin	100	0,49	0,01	0,53	0,00	0,16	NT
180	Gentamicin	1000	0,43	0,05	0,46	0,01	0,14	NT
181	Glucose	1	0,47	0,00	0,46	0,17	0,35	NT
182	Glucose	10	0,46	0,00	0,35	0,10	0,17	NT
183	Glucose	100	0,67	0,00	0,62	0,13	0,22	NT
184	Glucose	1000	0,74	0,00	0,68	0,19	0,28	NT
185	Glycochenodeoxycholate	1	0,08	0,00	0,18	0,00	0,15	MI, AP
186	Glycochenodeoxycholate	10	0,21	0,00	0,25	0,05	0,10	MI, AP
187	Glycochenodeoxycholate	100	0,06	0,00	0,34	0,00	0,00	MI, AP
188	Glycochenodeoxycholate	1000	0,77	0,08	0,90	0,26	0,19	MI, AP
189	Imipramine	1	0,08	0,00	0,20	0,00	0,00	MI, AP,CHOL
190	Imipramine	10	0,12	0,00	0,43	0,00	0,28	MI, AP,CHOL
191	Imipramine	100	0,57	0,72	0,19	0,00	0,30	MI, AP,CHOL
192	Imipramine	1000	0,73	0,62	0,40	0,55	0,60	MI, AP,CHOL
193	Indomethacin	1	0,50	0,00	0,23	0,00	0,36	
194	Indomethacin	10	0,50	0,13	0,38	0,08	0,32	
195	Indomethacin	100	0,51	0,00	0,48	0,00	0,21	
196	Indomethacin	1000	0,78	0,30	0,45	0,00	0,51	
197	Isoniazid	1	0,34	0,10	0,29	0,36	0,07	OS, MI
198	Isoniazid	10	0,29	0,05	0,40	0,37	0,08	OS, MI
199	Isoniazid	100	0,26	0,00	0,27	0,18	0,27	OS, MI
200	Isoniazid	1000	0,76	0,00	1,00	0,00	0,00	OS, MI
201	Ketoconazole	1	0,34	0,00	0,22	0,00	0,14	
202	Ketoconazole	10	0,33	0,00	0,30	0,43	0,07	
203	Ketoconazole	100	0,77	0,00	1,00	0,36	0,00	
204	Ketoconazole	1000	LP	LP	LP	LP	LP	
205	Ketotifen	1	0,74	0,19	0,57	0,02	0,15	NT
206	Ketotifen	10	0,71	0,27	0,44	0,22	0,29	NT
207	Ketotifen	100	0,90	0,31	0,47	0,17	0,40	NT
208	Ketotifen	1000	LP	LP	LP	LP	LP	NT
209	Lactose	1	0,54	0,05	0,04	0,00	0,00	NT
210	Lactose	10	0,90	0,23	0,00	0,00	0,00	NT
211	Lactose	100	0,47	0,17	0,00	0,13	0,04	NT
212	Lactose	1000	0,28	0,06	0,00	0,00	0,23	NT
213	Levofloxacin	1	0,47	0,00	0,50	0,26	0,13	OS, ST
214	Levofloxacin	10	0,57	0,00	0,56	0,29	0,17	OS, ST
215	Levofloxacin	100	0,65	0,00	0,43	0,35	0,04	OS, ST
216	Levofloxacin	1000	0,80	0,43	0,00	0,39	0,23	OS, ST
217	Lovastatin	1	0,39	0,13	0,33	0,09	0,07	OS
218	Lovastatin	10	0,68	0,22	0,33	0,09	0,16	OS
219	Lovastatin	100	0,79	0,30	0,51	0,14	0,53	OS
220	Lovastatin	1000	1,00	0,84	0,34	0,13	0,37	OS
221	Malathion	1	0,00	0,00	0,00	0,00	0,00	OS
222	Malathion	10	0,00	0,00	0,21	0,18	0,05	OS
223	Malathion	100	0,04	0,00	0,25	0,00	0,13	OS
224	Malathion	1000	0,15	0,00	0,41	0,00	0,01	OS
225	Maprotiline	1	0,34	0,15	0,50	0,00	0,32	MI
226	Maprotiline	10	0,39	0,24	0,35	0,01	0,10	MI
227	Maprotiline	100	0,79	0,54	0,48	0,31	0,41	MI
228	Maprotiline	1000	0,76	0,56	0,35	0,36	0,37	MI
229	Menadione	1	0,00	0,10	0,32	0,18	0,09	OS, MI, ST
230	Menadione	10	0,28	0,12	0,54	0,14	0,32	OS, MI, ST
231	Menadione	100	LP	LP	LP	LP	LP	OS, MI, ST
232	Menadione	1000	LP	LP	LP	LP	LP	OS, MI, ST
233	Mercaptopurine	1	0,29	0,00	0,27	0,14	0,06	OS, AP
234	Mercaptopurine	10	0,67	0,00	0,57	0,33	0,06	OS, AP
235	Mercaptopurine	100	0,62	0,20	0,54	0,48	0,33	OS, AP
236	Mercaptopurine	1000	0,77	0,28	0,27	0,53	0,09	OS, AP
237	Mercury II	1	0,50	0,09	0,10	0,01	0,13	OS, MI, AP
238	Mercury II	10	0,84	0,34	0,33	0,00	0,02	OS, MI, AP
239	Mercury II	100	0,00	0,51	0,00	0,42	0,00	OS, MI, AP
240	Mercury II	1000	0,85	0,98	0,01	0,00	0,00	OS, MI, AP
241	Methotrexate	1	0,51	0,11	0,23	0,41	0,27	OS
242	Methotrexate	10	0,51	0,34	0,00	0,82	0,10	OS
243	Methotrexate	100	0,52	0,05	0,12	0,61	0,18	OS
244	Methotrexate	1000	0,94	0,34	0,07	0,55	0,08	OS
245	N-acetylcysteine	1	0,54	0,20	0,49	0,01	0,03	NT
246	N-acetylcysteine	10	0,51	0,15	0,34	0,08	0,02	NT
247	N-acetylcysteine	100	0,40	0,16	0,44	0,12	0,03	NT
248	N-acetylcysteine	1000	0,37	0,16	0,27	0,00	0,00	NT
249	NNK	1	0,40	0,21	0,08	0,31	0,20	
250	NNK	10	0,55	0,36	0,15	0,31	0,08	
251	NNK	100	0,48	0,25	0,19	0,08	0,38	
252	NNK	1000	0,51	0,28	0,33	0,18	0,37	
253	Orphenadrine	1	0,32	0,15	0,46	0,00	0,18	
254	Orphenadrine	10	0,18	0,19	0,11	0,00	0,00	
255	Orphenadrine	100	0,63	0,14	0,52	0,00	0,20	
256	Orphenadrine	1000	LP	LP	LP	LP	LP	
257	Paraquat	1	0,00	0,00	0,41	0,00	0,22	OS
258	Paraquat	10	0,18	0,00	0,78	0,00	0,29	OS
259	Paraquat	100	0,00	0,00	0,93	0,00	1,00	OS
260	Paraquat	1000	0,25	0,16	0,77	0,00	0,87	OS
261	Pentachlorophenol	1	0,55	0,15	0,64	0,04	0,29	OS
262	Pentachlorophenol	10	0,02	0,00	0,21	0,00	0,00	OS
263	Pentachlorophenol	100	0,00	0,10	0,39	0,00	0,16	OS
264	Pentachlorophenol	1000	0,60	0,35	0,27	0,43	0,30	OS
265	Phenobarbital	1	0,28	0,15	0,50	0,19	0,18	MI
266	Phenobarbital	10	0,05	0,04	0,39	0,13	0,15	MI
267	Phenobarbital	100	0,08	0,16	0,11	0,14	0,00	MI

order	Compound	Concentration (μM)	TOX Index	OS Index	MI Index	APT Index	ST Index	Bibliography Mechanism
268	Phenobarbital	1000	0,00	0,03	0,25	0,21	0,00	MI
269	Phenytoin	1	0,46	0,00	0,53	0,00	0,55	MI
270	Phenytoin	10	0,44	0,10	0,28	0,09	0,42	MI
271	Phenytoin	100	1,00	0,36	1,00	0,00	1,00	MI
272	Phenytoin	1000	1,00	0,59	1,00	0,00	0,95	MI
273	Piperonylbutoxide	1	0,38	0,16	0,31	0,07	0,00	AP
274	Piperonylbutoxide	10	0,36	0,08	0,44	0,00	0,00	AP
275	Piperonylbutoxide	100	0,88	0,00	1,00	0,00	0,27	AP
276	Piperonylbutoxide	1000	0,80	0,00	1,00	0,00	0,52	AP
277	Propanolol	1	0,54	0,20	0,41	0,19	0,41	
278	Propanolol	10	0,67	0,13	0,26	0,07	0,41	
279	Propanolol	100	0,92	0,49	0,29	0,09	0,60	
280	Propanolol	1000	1,00	1,00	0,35	0,24	1,00	
281	Riboflavin	1	0,37	0,04	0,19	0,15	0,28	
282	Riboflavin	10	0,56	0,12	0,44	0,13	0,52	
283	Riboflavin	100	0,55	0,06	0,20	0,07	0,52	
284	Riboflavin	1000	0,92	0,52	0,00	0,56	0,53	
285	Rifampicin	1	0,50	0,08	0,08	0,23	0,16	OS, MI
286	Rifampicin	10	0,39	0,00	0,39	0,00	0,37	OS, MI
287	Rifampicin	100	0,80	0,19	0,64	0,00	0,35	OS, MI
288	Rifampicin	1000	0,72	0,93	0,00	0,49	0,17	OS, MI
289	Simvastatin	1	0,45	0,23	0,14	0,09	0,19	MI, AP
290	Simvastatin	10	0,49	0,32	0,28	0,11	0,42	MI, AP
291	Simvastatin	100	0,78	0,57	0,25	0,18	0,38	MI, AP
292	Simvastatin	1000	0,67	0,67	0,00	0,40	0,20	MI, AP
293	Sorbitol	1	0,09	0,11	0,32	0,17	0,17	NT
294	Sorbitol	10	0,19	0,02	0,46	0,28	0,16	NT
295	Sorbitol	100	0,14	0,05	0,26	0,20	0,12	NT
296	Sorbitol	1000	0,14	0,02	0,43	0,00	0,25	NT
297	Stavudine	1	0,41	0,09	0,32	0,15	0,23	MI
298	Stavudine	10	0,56	0,22	0,40	0,29	0,27	MI
299	Stavudine	100	0,72	0,28	0,43	0,24	0,23	MI
300	Stavudine	1000	0,70	0,31	0,34	0,35	0,33	MI
301	Tamoxifen	1	0,22	0,00	0,00	0,02	0,00	OS, MI, ST
302	Tamoxifen	10	0,60	0,15	0,29	0,00	0,21	OS, MI, ST
303	Tamoxifen	100	0,51	0,00	0,33	0,01	0,45	OS, MI, ST
304	Tamoxifen	510	LP	LP	LP	LP	LP	OS, MI, ST
305	Taurolithocholate	1	0,51	0,22	0,16	0,10	0,19	OS, AP, ST
306	Taurolithocholate	10	0,75	0,27	0,50	0,14	0,25	OS, AP, ST
307	Taurolithocholate	100	0,68	0,27	0,34	0,21	0,21	OS, AP, ST
308	Taurolithocholate	1000	0,82	0,39	0,16	0,28	0,08	OS, AP, ST
309	Tetracycline	1	0,31	0,18	0,23	0,02	0,23	MI, ST
310	Tetracycline	10	0,45	0,25	0,30	0,23	0,15	MI, ST
311	Tetracycline	100	0,55	0,48	0,25	0,00	0,46	MI, ST
312	Tetracycline	1000	0,78	0,51	0,37	0,12	0,42	MI, ST
313	Thiabendazole	1	0,61	0,23	0,32	0,11	0,17	CHOL
314	Thiabendazole	10	0,44	0,16	0,32	0,08	0,09	CHOL
315	Thiabendazole	100	0,70	0,23	0,48	0,09	0,17	CHOL
316	Thiabendazole	1000	1,00	0,39	0,42	0,15	0,10	CHOL
317	Thiamine	1	0,65	0,12	0,49	0,21	0,42	NT
318	Thiamine	10	0,71	0,08	0,51	0,20	0,33	NT
319	Thiamine	100	0,62	0,00	0,36	0,07	0,29	NT
320	Thiamine	1000	0,65	0,13	0,19	0,19	0,23	NT
321	Thioacetamide	1	0,56	0,16	0,61	0,00	0,03	OS, ST
322	Thioacetamide	10	0,58	0,16	0,50	0,04	0,07	OS, ST
323	Thioacetamide	100	0,41	0,16	0,37	0,06	0,14	OS, ST
324	Thioacetamide	1000	0,36	0,02	0,29	0,00	0,27	OS, ST
325	Ticlopidine	1	0,50	0,26	0,20	0,06	0,12	OS
326	Ticlopidine	10	0,60	0,33	0,07	0,13	0,17	OS
327	Ticlopidine	100	1,00	0,34	0,65	0,08	0,19	OS
328	Ticlopidine	1000	0,96	0,37	0,57	0,63	0,11	OS
329	Tilorone	1	0,69	0,10	0,46	0,30	0,12	OS
330	Tilorone	10	0,88	0,17	0,58	0,00	0,98	OS
331	Tilorone	100	0,92	0,42	0,17	0,23	0,44	OS
332	Tilorone	1000	LP	LP	LP	LP	LP	OS
333	Troglitazone	1	0,52	0,16	0,27	0,17	0,09	MI, AP,CHOL
334	Troglitazone	10	0,62	0,23	0,22	0,17	0,11	MI, AP,CHOL
335	Troglitazone	100	0,53	0,36	0,00	0,91	0,00	MI, AP,CHOL
336	Troglitazone	500	0,82	0,77	0,05	0,46	0,25	MI, AP,CHOL
337	Valproic	1	0,73	0,18	0,67	0,00	0,01	MI, ST
338	Valproic	10	0,43	0,09	0,66	0,02	0,01	MI, ST
339	Valproic	100	0,52	0,16	0,63	0,05	0,00	MI, ST
340	Valproic	1000	0,65	0,23	0,35	0,00	0,00	MI, ST
341	Verapamil	1	0,00	0,03	0,35	0,03	0,14	
342	Verapamil	10	0,53	0,30	0,49	0,36	0,38	
343	Verapamil	100	1,00	0,27	0,87	0,00	0,44	
344	Verapamil	1000	LP	LP	LP	LP	LP	
345	Warfarin	1	0,27	0,02	0,47	0,00	0,00	MI
346	Warfarin	10	0,26	0,07	0,50	0,05	0,00	MI
347	Warfarin	100	0,40	0,11	0,35	0,15	0,00	MI
348	Warfarin	1000	0,77	0,00	0,84	0,10	0,32	MI
